# EBBP‐Mediated Integrated Stress Response Attenuates Anthracycline‐Induced Cardiotoxicity by Inhibiting the Ferroptosis of Cardiomyocytes

**DOI:** 10.1002/advs.202502726

**Published:** 2025-06-10

**Authors:** Zilong Chen, Can Chen, Yichen Wu, Yinxue Xia, Ruijie Luo, Jiangcheng Shu, Long Chen, Zhaohui Wang, Cheng Wang, Kai Huang

**Affiliations:** ^1^ Department of Cardiology Union Hospital Tongji Medical College Huazhong University of Science and Technology Wuhan 430022 China; ^2^ Clinic Center of Human Genomic Research Union Hospital Tongji Medical College Huazhong University of Science and Technology Wuhan 430022 China; ^3^ Department of Geriatrics Southwest Hospital Third Military Medical University (Army Medical University) Chongqing 400038 China; ^4^ Department of Rheumatology Union Hospital Tongji Medical College Huazhong University of Science and Technology Wuhan 430022 China; ^5^ Hubei Key Laboratory of Metabolic Abnormalities and Vascular Aging Huazhong University of Science and Technology Wuhan 430022 China; ^6^ Hubei Clinical Research Center of Metabolic and Cardiovascular Disease Huazhong University of Science and Technology Wuhan 430022 China

**Keywords:** anthracycline‐induced cardiotoxicity, EBBP, ferroptosis, K63‐linked ubiquitination, PERK‐mediated integrated stress response

## Abstract

Anthracyclines are potent chemotherapeutics, but their clinical application is constrained by dose‐dependent cardiotoxicity, in which ferroptosis plays a critical role. Here, EBBP (Estrogen‐responsive B Box Protein) is identified as a key cardioprotective regulator in anthracycline‐induced cardiotoxicity. Transcriptomic profiling of doxorubicin (DOX)‐treated hearts reveals significant EBBP upregulation. Cardiac‐specific overexpression of EBBP protects against myocardial injury and dysfunction by reducing DOX‐induced ferroptosis. Conversely, EBBP silencing exacerbates DOX‐induced cardiac damage, an effect reversed by ferroptosis inhibitor ferrostatin‐1 (Fer‐1). The molecular targets of EBBP are subsequently identified through bulk RNA sequencing, molecular docking analysis, co‐immunoprecipitation experiments, and ubiquitination assays. Mechanistically, EBBP interacts with GRP78 to promote its K63‐linked ubiquitination, disrupting the inhibitory GRP78‐PERK interaction and activating PERK‐mediated integrated stress response (ISR). This signaling cascade ultimately leads to the activation of downstream effectors ATF4 and Nrf2, which coordinately upregulates the SLC7A11/GSH/GPX4 axis and restores iron homeostasis. Importantly, pharmacological inhibition of PERK abolishes the protective effects of EBBP against myocardial injury and ferroptosis. Overall, our findings identify EBBP as a novel suppressor of ferroptosis in anthracycline‐induced cardiotoxicity via the PERK‐mediated ISR, thereby underscoring its therapeutic potential for preventing anthracycline‐induced cardiomyopathy.

## Graphical abstract

EBBP expression is significantly upregulated in DOX‐treated cardiomyocytes. Mechanistically, EBBP interacts with GRP78 to mediate K63‐linked ubiquitination, thereby attenuating the inhibitory GRP78‐PERK interaction and triggering activation of the PERK‐mediated integrated stress response (ISR). This signaling cascade culminates in the activation of downstream effectors ATF4 and Nrf2, which transcriptionally upregulate the SLC7A11‐GSH‐GPX4 antioxidant axis and concurrently alleviate iron overload.

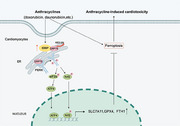

## 1 Introduction

In recent years, significant advancements in early cancer detection, medical technologies, and therapeutic strategies have led to a marked decline in cancer mortality.^[^ ^1^ ^]^ However, these clinical benefits are often counterbalanced by severe treatment‐related side effects, particularly cardiovascular complications.^[^ ^2^, ^3^ ^]^ Among chemotherapeutic agents, anthracyclines—especially doxorubicin (DOX)—remain a cornerstone in treating both solid and hematological malignancies.^[^ ^4^, ^5^ ^]^ Despite their efficacy, chronic anthracycline use induces dose‐dependent and frequently irreversible cardiotoxicity, manifesting as arrhythmias, acute cardiac dysfunction, cardiomyopathy, and heart failure.^[^ ^6^, ^7^, ^8^ ^]^ The pathogenesis of anthracycline‐induced cardiotoxicity is multifactorial, involving oxidative stress, mitochondrial dysfunction, impaired autophagy, inflammatory responses, and various forms of cell death.^[^ ^9^, ^10^ ^]^ To improve patient outcomes, a deeper understanding of the underlying mechanisms and the identification of novel therapeutic targets are urgently needed.

Emerging evidence underscores ferroptosis, an iron‐dependent form of regulated cell death driven by lipid peroxidation, as a critical contributor to DOX‐induced cardiomyopathy.^[^ ^11^, ^12^ ^]^ Anthracyclines and their metabolites disrupt iron homeostasis by altering the expression of key regulatory proteins, including transferrin receptor (TfR), ferritin, and ferroportin (FPN).^[^ ^13^, ^14^ ^]^ Concurrently, they deplete antioxidant defenses, such as glutathione peroxidase 4 (GPX4) and glutathione (GSH), thereby exacerbating lipid peroxidation and driving cell death.^[^ ^15^ ^]^ Preclinical studies reveal that ferroptosis inhibitors (e.g., ferrostatin‐1) and iron chelators (e.g., deferoxamine and dexrazoxane) mitigate DOX‐induced cardiac injury, highlighting ferroptosis as a promising therapeutic target.^[^ ^16^, ^17^, ^18^ ^]^ However, the clinical translation of these agents is limited by poor stability and specificity. Moreover, since ferroptosis inhibition may compromise anthracycline's antitumor efficacy, identifying tissue‐specific regulators of ferroptosis is imperative to selectively protect the heart without compromising tumor sensitivity.

The integrated stress response (ISR) is an adaptive cellular mechanism that enables organisms to respond to diverse stress conditions, including endoplasmic reticulum (ER) stress, glucose and amino acid deprivation, hypoxia, viral infection, and oxidative stress mediated by reactive oxygen species (ROS).^[^ ^19^ ^]^ Upon activation of the ISR, four upstream kinases—HRI (heme‐regulated inhibitor kinase), PKR (interferon‐induced double‐stranded RNA‐activated protein kinase), PERK (PKR‐like ER kinase), and GCN2 (general control nonderepressible 2)—phosphorylate eIF2α, thereby globally attenuating protein synthesis. Simultaneously, key ISR effectors, such as activating transcription factor 4 (ATF4), are selectively upregulated to enhance protein‐folding capacity and alleviate oxidative stress.^[^ ^20^, ^21^ ^]^ Previous studies have demonstrated that the ISR plays a crucial role in maintaining cardiac homeostasis. PKR deficiency confers cardioprotection against hemodynamic stress.^[^ ^22^ ^]^ Similarly, GCN2 knockout enhances cardiac functional recovery following pressure overload.^[^ ^23^ ^]^ Unlike the other three kinases, activation of the PERK branch has been shown to suppress myocardial ischemia‐reperfusion injury and pressure‐induced hypertrophy, suggesting a protective role in cardiac pathophysiology.^[^ ^24^, ^25^ ^]^ Notably, accumulating evidence indicates that the ISR plays a pivotal role in modulating ferroptosis. Specifically, it has been observed that the PERK/ATF4 pathway inhibits ferroptosis by regulating solute carrier family 7 member 11 (SLC7A11).^[^ ^26^ ^]^ Optic atrophy 1 (OPA1) has been shown to promote ferroptosis by suppressing ISR.^[^ ^27^ ^]^ However, the precise role of ISR in anthracycline‐induced cardiomyopathy warrants further investigation.

To identify novel therapeutic targets, we analyzed differentially expressed genes in a murine model of DOX‐induced cardiomyopathy and discovered that EBBP, an E3 ubiquitin ligase, was significantly upregulated.^[^ ^28^ ^]^ EBBP is known to regulate autophagy, lysosomal function, and cellular homeostasis,^[^ ^29^ ^]^ and has been implicated in mitigating lipid deposition and inflammation in non‐alcoholic steatohepatitis (NASH).^[^ ^30^ ^]^ Additionally, EBBP exhibits cardioprotective effects in pathological hypertrophy and ischemia/reperfusion injury.^[^ ^31^, ^32^ ^]^ But its role in anthracycline‐induced cardiotoxicity remains to be further investigated.

In this study, we systematically investigated the role of EBBP in doxorubicin‐induced cardiotoxicity (DoIC) using both in vivo and in vitro models. Mechanistically, we found that EBBP enhances the K63‐linked polyubiquitination of GRP78, attenuating its inhibitory interaction with PERK and triggering activation of the PERK‐mediated ISR pathway. This signaling cascade ultimately leads to the activation of downstream effectors ATF4 and Nrf2, which transcriptionally upregulate the SLC7A11‐GSH‐GPX4 antioxidant axis while alleviating iron overload. Our findings not only establish EBBP as a novel regulator of ferroptosis but also highlight its potential as a therapeutic target for mitigating anthracycline‐induced cardiotoxicity.

## 2 Results

### 2.1 EBBP is Significantly Upregulated in Anthracycline‐Induced Cardiotoxicity

**Figure 1:**
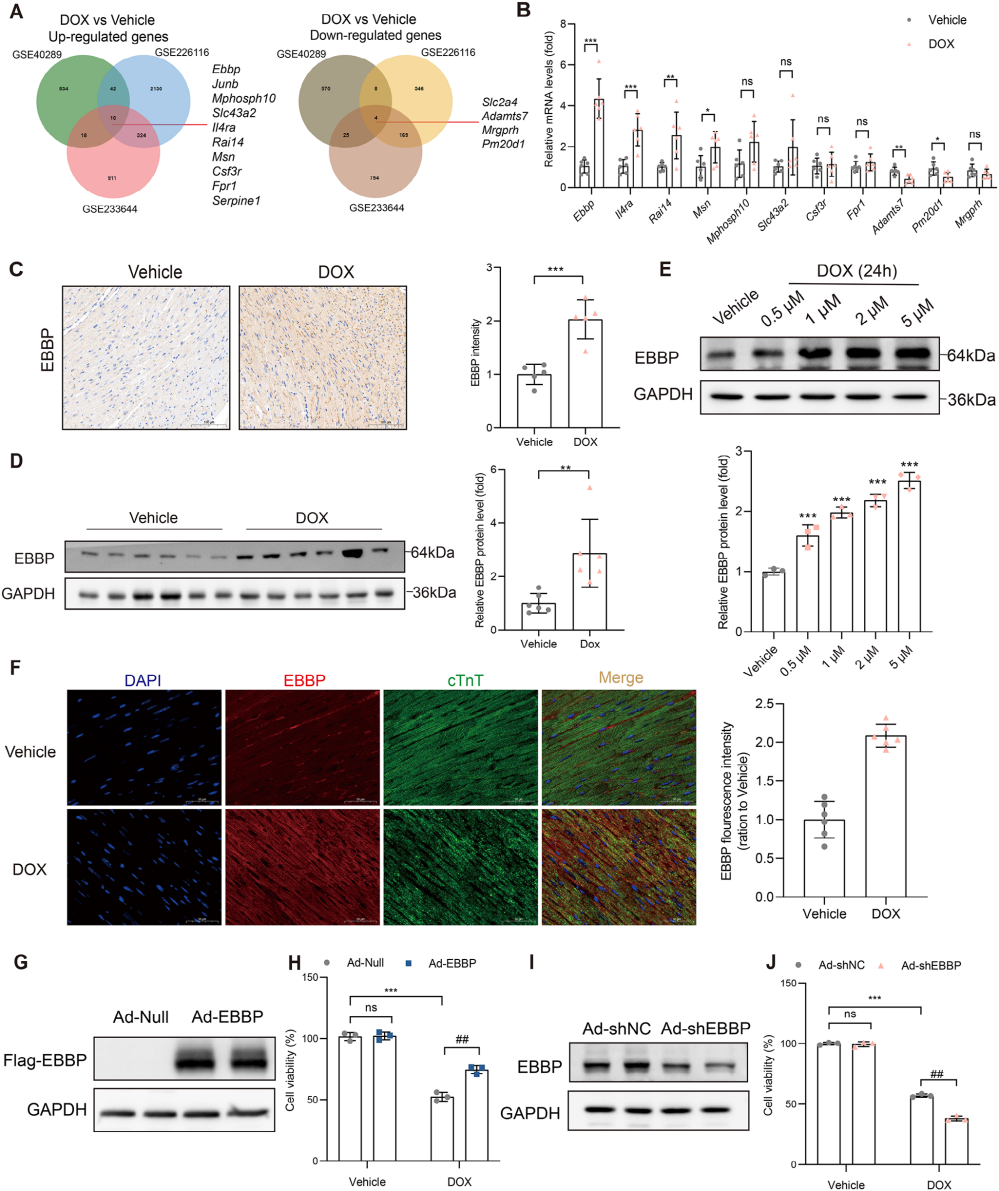
EBBP is significantly upregulated in anthracycline‐induced cardiotoxicity. A) Venn diagram showing differentially expressed genes that are consistently upregulated or downregulated across all three GEO datasets (GSE40289, GSE226116, GSE233644, DOX vs Vehicle). B) The mRNA levels of candidate genes in cardiac tissues of mice treated with DOX (*n* = 6). C) Immunohistochemistry and statistical analysis of EBBP expression in the myocardium of DOX‐treated mice (scale bar, 100 µm). D) Immunoblots and statistical analysis of EBBP protein level in the heart of mice treated with DOX (*n* = 6). E) Immunoblots and statistical analysis of EBBP expression in NRCMs treated with different concentrations of doxorubicin for 24 h (*n* = 3). F) Representative immunofluorescence staining of EBBP (red) and cTnT (green) in mouse heart tissue. Nuclei were stained with DAPI (blue). (scale bars, 50 µm; *n* = 6). Statistical analyses of EBBP mean fluorescence intensity are presented in the panel on the right. G,I) Adenovirus‐mediated EBBP overexpression or depletion in vitro was assessed by western blot analysis. H,J) Following infection with Ad‐EBBP or Ad‐shEBBP, H9c2 was subjected to a DOX (1 µm) treatment for a duration of 24 h. Cell viability was measured by CCK8 (*n* = 3). Values are presented as the mean ± SD. *p < 0.05, **p < 0.01 and ***p < 0.001 vs Vehicle group; ^##^ *p* < 0.01 vs DOX+ Ad‐Null or DOX+ Ad‐shNC.

To identify the key genes that play a critical role in anthracycline‐induced cardiotoxicity, we systematically analyzed RNA sequencing data of the doxorubicin‐induced cardiomyopathy model obtained from the Gene Expression Omnibus (GEO) database (GSE40289, GSE226116, GSE233644). By employing a fold change threshold exceeding 1.5 and a statistically significant *p*‐value cutoff (<0.05), our analysis revealed 10 consistently upregulated genes across all three datasets, as well as 4 downregulated genes (**Figure** **1**A). While *Junb*, *Serpine1*, and *Slc2a4* have been previously reported to be associated with anthracycline‐induced cardiotoxicity,^[^ ^33^, ^34^, ^35^ ^]^ subsequent validation in our DOX‐induced cardiomyopathy (DoIC) mouse model revealed EBBP as the most significantly altered gene among the remaining candidates (*Ebbp*, *Mphosph10*, *Slc43a2*, *Il4ra*, *Rai14*, *Msn*, *Csf3r*, *Fpr1*, *Adamts7*, *Mrgprh*, *Pm20d1*) (Figure 1B).

Consistent with the transcriptomic findings, immunohistochemical analysis demonstrated markedly increased EBBP expression in DOX‐treated myocardium compared to controls (Figure 1C). Western blot analysis further confirmed the upregulation of EBBP protein levels in cardiac tissues following DOX treatment (Figure 1D). In cultured cardiomyocytes, both mRNA and protein expression of EBBP showed dose‐ and time‐dependent increases upon DOX exposure (Figure 1E; Figure S1A–C, Supporting Information), suggesting a direct relationship between DOX treatment and EBBP induction.

Immunofluorescence studies revealed specific co‐localization of EBBP with cardiac troponin T (cTnT), a specific marker for cardiomyocytes, confirming its predominant expression and elevated levels in cardiomyocytes under DOX‐induced stress conditions (Figure 1F). This cardiomyocyte‐specific expression pattern was further validated in neonatal rat cardiomyocytes (NRCMs) (Figure S1D, Supporting Information), supporting the notion that EBBP upregulation represents a specific response of cardiomyocytes to anthracycline toxicity.

To investigate the molecular mechanisms underlying doxorubicin‐induced EBBP upregulation, the putative upstream transcription factors of EBBP were identified by JASPAR, CHEA, GTRD, and ChIP‐Atlas databases (Figure S1E, Supporting Information). Several candidate transcription factors were selected for further validation. Notably, luciferase reporter assays revealed that ZNF384 most potently enhanced EBBP promoter activity compared to other tested transcription factors, indicating that ZNF384 likely serves as a key transcriptional regulator of EBBP (Figure S1F, Supporting Information).

### 2.2 EBBP Protects Against Anthracycline‐Induced Cardiomyocyte Damage In Vitro

To determine the role of EBBP in doxorubicin‐induced cardiomyocyte injury, we employed adenoviral‐mediated gene manipulation approaches. Cardiomyocytes were transduced with either EBBP‐overexpressing or EBBP‐knockdown vectors prior to DOX treatment (Figure 1G,I). The results indicate that DOX treatment markedly decreased cell viability, whereas overexpression of EBBP significantly improved cell viability (Figure 1H). Conversely, the knockdown of EEBP further exacerbated the decrease in cell viability (Figure 1J). Furthermore, TUNEL staining revealed that EBBP overexpression significantly attenuated DNA damage (Figure S1G, Supporting Information), whereas EBBP knockdown exacerbated DNA damage (Figure S1H, Supporting Information). Collectively, these findings demonstrate that EBBP attenuates DOX‐induced cardiomyocyte injury in vitro.

### 2.3 EBBP Overexpression Ameliorates Anthracycline‐Induced Cardiac Injury

**Figure 2:**
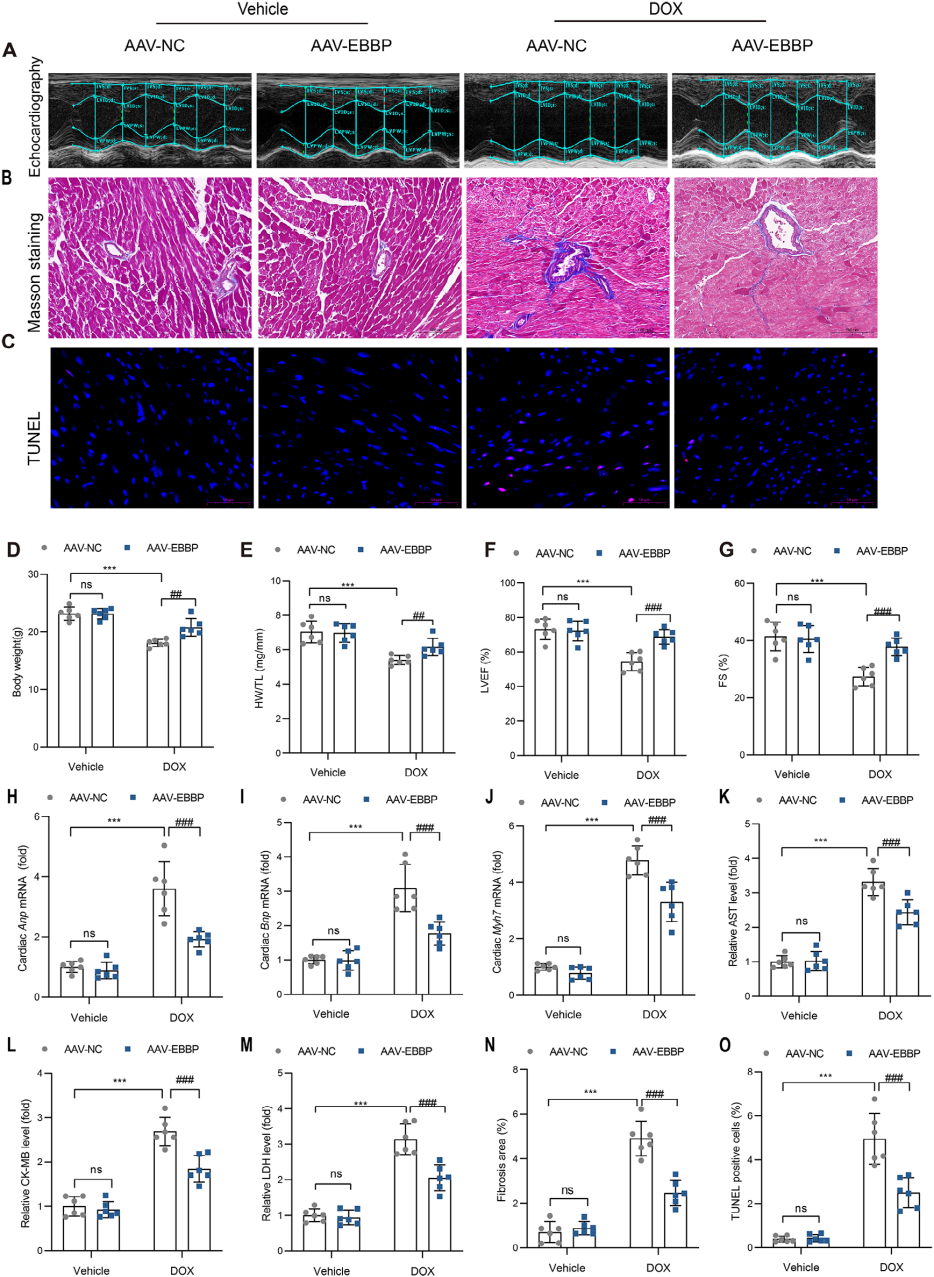
EBBP overexpression ameliorates anthracycline‐induced cardiac injury. AAV‐NC or AAV‐EBBP was administered via tail vein injection, and the DoIC model was established 14 days thereafter. A) Representative images of transthoracic echocardiography. B) Representative images of Masson's trichrome staining (scale bars, 100 µm). C) Representative images of TUNEL staining (scale bars, 50 µm). D) Changes in body weight of mice after DOX injection (*n* = 6). E) Changes in HW/TL (*n* = 6). F,G) Statistical analysis of LVEF and FS (*n* = 6). H–J) The mRNA levels of Anp, Bnp, and Myh7 in cardiac tissues (*n* = 6). K–M) Statistical analysis of serum AST, CK‐MB, and LDH levels (*n* = 6). N) Statistical analysis of fibrosis area (*n* = 6). O) Quantitative analysis of TUNEL‐positive cells via immunofluorescence staining (*n* = 6). Values are presented as the mean ± SD. ***p < 0.001 vs Vehicle + AAV‐NC; ^##^ *p* < 0.01 and ^###^ *p* < 0.001 vs DOX + AAV‐NC group.

To elucidate the effect of EBBP on DoIC, AAV9 was administered via tail vein injection to establish the model of cardiomyocyte‐specific EBBP overexpression (Figure S2A,B, Supporting Information). Two weeks following viral administration, DOX was intraperitoneally injected into mice. DOX treatment significantly reduced both body weight (BW) and the heart weight‐to‐tibial length ratio (HW/TL). Notably, these adverse effects were substantially mitigated by the cardiomyocyte‐specific overexpression of EBBP (**Figure** **2**D,E). Echocardiographic assessments (Figure 2A) indicated that cardiac function improved in the EBBP overexpression group (AAV‐EBBP) following DOX injection, as demonstrated by elevated left ventricular ejection fraction (LVEF) (Figure 2F) and fractional shortening (FS) (Figure 2G). Correspondingly, the mRNA levels of heart failure biomarkers, including atrial natriuretic peptide (*Anp*), brain natriuretic peptide (*Bnp*), and myosin‐7 (*Myh7*), were markedly lower in the DOX‐treated EBBP‐overexpressing mice (Figure 2H–J). Furthermore, serum analysis showed that EBBP overexpression markedly suppressed DOX‐induced elevations in cardiac injury biomarkers AST, CK‐MB, and LDH (Figure 2K–M).

Histopathological examination revealed that EBBP overexpression significantly ameliorated DOX‐induced myocardial structural abnormalities, including the disorganized arrangement of cardiomyocytes and loss of characteristic striations (Figure S2D, Supporting Information). Masson's trichrome staining demonstrated that EBBP overexpression effectively reduced DOX‐mediated cardiac fibrosis (Figure 2B,N). Furthermore, TUNEL assays showed that EBBP overexpression significantly decreased DOX‐induced DNA fragmentation in myocardial tissue (Figure 2C,O). Importantly, while DOX treatment markedly increased myocardial pro‐inflammatory cytokine levels, this effect was substantially attenuated by EBBP overexpression (Figure S2E, Supporting Information). Collectively, these findings suggest that EBBP exerts protective effects against DoIC in murine models.

### 2.4 EBBP Deficiency Aggravates Anthracycline‐Induced Cardiac Injury

**Figure 3:**
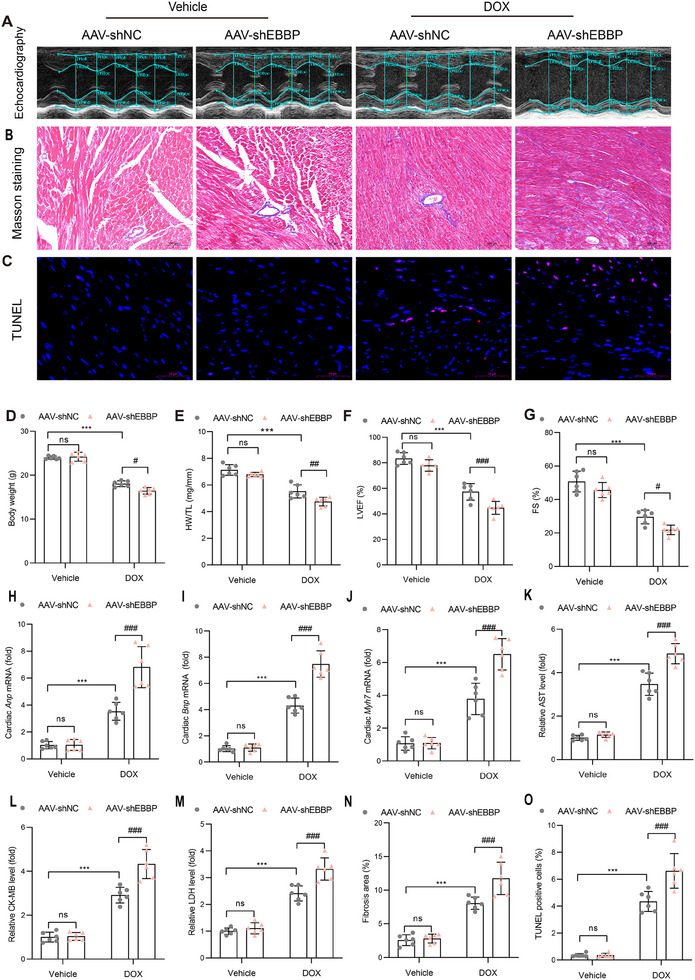
EBBP deficiency aggravates anthracycline‐induced cardiac injury. AAV‐shNC or AAV‐shEBBP was administered via tail vein injection, and the DoIC model was established 14 days thereafter. A) Representative images of transthoracic echocardiography. B) Representative images of Masson's trichrome staining (scale bars, 100 µm). C) Representative images of TUNEL staining (scale bars, 50 µm). D) Changes in body weight of mice after DOX injection (*n* = 6). E) Changes in HW/TL (*n* = 6). F,G) Statistical analysis of LVEF and FS (*n* = 6). H–J) The mRNA levels of Anp, Bnp, and Myh7 in cardiac tissues (*n* = 6). K–M) Statistical analysis of serum AST, CK‐MB, and LDH levels (*n* = 6). N) Statistical analysis of fibrosis area (*n* = 6). O) Quantitative analysis of TUNEL‐positive cells via immunofluorescence staining (*n* = 6). Values are presented as the mean ± SD. ***p < 0.001 vs Vehicle + AAV‐shNC; ^#^ *p* < 0.05, ^##^ *p* < 0.01 and ^###^ *p* < 0.001 vs DOX + AAV‐ shNC group.

To further investigate the role of EBBP in anthracycline‐induced cardiomyopathy, AAV‐shEBBP or AAV‐shNC was administered to modulate the expression levels of EBBP prior to doxorubicin treatment (Figure S2C, Supporting Information). Our findings indicated that the knockdown of EBBP exacerbated DOX‐induced reductions in body weight and HW/TL (**Figure** **3**D,E). Echocardiographic assessments revealed that EBBP knockdown further deteriorated DOX‐induced cardiac dysfunction, as demonstrated by decreased LVEF and FS (Figure 3A,F,G). Moreover, mRNA levels of heart failure markers (*Anp*, *Bnp*, and *Myh7*) were markedly elevated in DOX‐treated AAV‐shEBBP mice compared to DOX‐treated AAV‐shNC controls (Figure 3H–J). The DOX‐induced increase of serum AST, CK‐MB, and LDH was also further aggravated by EBBP knockdown (Figure 3K–M).

Histological analysis further demonstrated that EBBP knockdown intensified DOX‐induced disorganization of myofibrillar structures (Figure S2F, Supporting Information) and promoted excessive cardiac collagen deposition (Figure 3B,N). TUNEL staining also revealed that EBBP deficiency exacerbated DOX‐induced DNA fragmentation in the myocardium (Figure 3C,O). Furthermore, the production of pro‐inflammatory cytokines in the myocardium was significantly amplified by EBBP knockdown (Figure S2G, Supporting Information). These results demonstrate that EBBP deficiency worsens DOX‐induced cardiotoxicity, confirming its essential cardioprotective role during anthracycline treatment.

### 2.5 EBBP Ameliorates Anthracycline‐Induced Cardiomyocyte Ferroptosis

**Figure 4:**
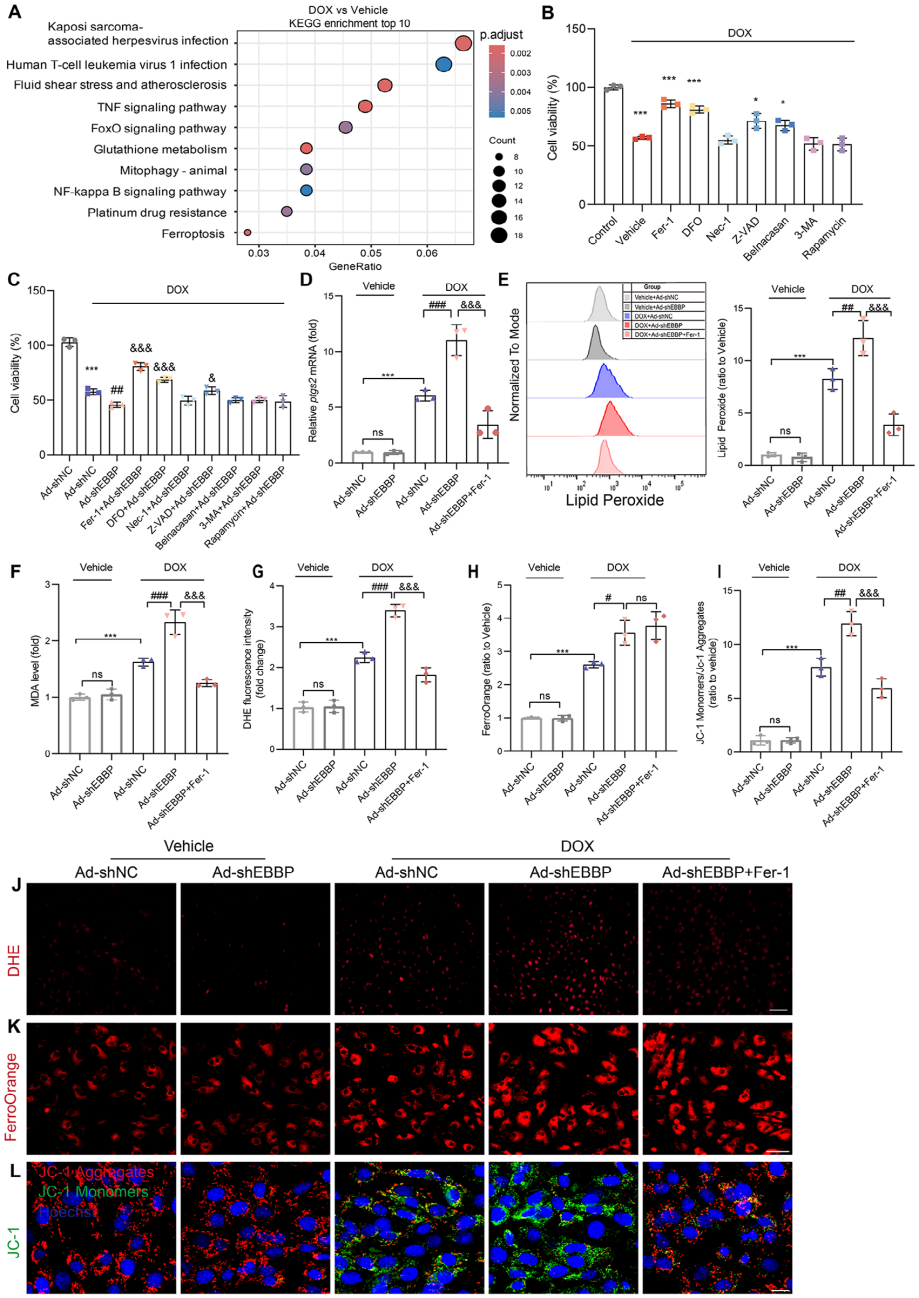
EBBP deficiency aggravates anthracycline‐induced cardiomyocyte ferroptosis in vitro. A) KEGG pathway enrichment analysis of the GEO dataset (GSE233644) comparing DOX‐treated murine hearts with control murine hearts. B) The effects of various cell death inhibitors on the viability of H9c2 cells treated with DOX (1 µm) (*n* = 3). C) The effects of various cell death inhibitors on the viability of H9c2 cells transduced with Ad‐shEBBP and treated with DOX (1 µm) (n=3). D–L) Following infection with Ad‐shNC or Ad‐shEBBP, H9c2 was pretreated with Fer‐1 (2 µm) for 2h and then treated with DOX (1 µm) for 24 h. D) The mRNA level of Ptgs2 was detected by qRT‐PCR (*n* = 3). E) Representative pictures and statistical analysis of intracellular lipid peroxide by flow cytometry (*n* = 3). F) MDA level in cells (*n* = 3). G) Quantification of fluorescent immunohistochemistry staining for DHE in H9c2 cells (*n* = 3). H) Quantification of intracellular Fe^2+^ levels (FerroOrange staining) in H9c2 cells (*n* = 3). I) Statisticaof mitochondrial membrane potential (JC‐1 staining) in H9c2 cells (scale bars, 20 µm; *n* = 3). J–L) Representative images of DHE staining (scale bars, 100 µm), FerroOrange staining (scale bars, 50 µm), and JC‐1 staining (scale bars, 20 µm). Values are presented as the mean ± SD. ***p < 0.001 vs Vehicle +Ad‐shNC; ^#^ *p* < 0.05, ^##^ *p* < 0.01 and ^###^ *p* < 0.001 vs DOX+ Ad‐shNC; ^&&&^ *p* < 0.001 vs DOX+Ad‐shEBBP group.

To characterize the molecular pathways involved in anthracycline‐induced cardiomyopathy, we performed Kyoto Encyclopedia of Genes and Genomes (KEGG) pathway enrichment analysis on GEO transcriptomic data (GSE233644). Here, we found that ferroptosis, NF‐kappa B pathway, mitophagy, glutathione metabolism, Foxo, and TNF signaling pathways were significantly enriched (**Figure** **4**A). These pathways are mechanistically associated with distinct cell death, indicating that multiple forms of programmed cell death (e.g., apoptosis, ferroptosis, autophagy, necroptosis, pyroptosis) underlie the pathogenesis of doxorubicin‐induced cardiomyopathy. Then, a panel of specific inhibitors was employed to investigate the precise mechanisms underlying DOX‐mediated cell death. Among these, 3‐MA (an autophagy inhibitor), rapamycin (an autophagy stimulator), and Nec‐1 (a necroptosis inhibitor) failed to inhibit DOX‐induced cell death. However, Fer‐1 (a ferroptosis inhibitor), DFO (an iron chelator), ZVAD‐FMK (an apoptosis inhibitor), and belnacasan (a pyroptosis inhibitor) effectively attenuated DOX‐induced cell death, with Fer‐1 and DFO exhibiting the most pronounced effects (Figure 4B). Collectively, these findings indicate that ferroptosis may serve as the predominant cell death mechanism under DOX stimulation, which is consistent with previous studies.^[^ ^16^, ^36^ ^]^

We further investigated the molecular mechanisms underlying EBBP‐mediated protection against anthracycline‐induced cardiotoxicity. RNA sequencing of DOX‐treated EBBP‐overexpressing cardiac tissues (|FC|≥2, *p* < 0.05) identified 187 upregulated and 143 downregulated genes (Figure S3A, Supporting Information). KEGG pathway enrichment analysis of differentially expressed genes (DEGs) also demonstrated significant enrichment in ferroptosis‐related pathways (Figure S3B, Supporting Information). Subsequently, we found that ferroptosis inhibitors (Fer‐1 and DFO) exerted the most significant inhibitory effect on cell death exacerbated by EBBP knockdown compared to other inhibitors (Figure 4C). Correspondingly, western blot analysis confirmed that EBBP did not modulate key proteins involved in necroptosis, autophagy, or pyroptosis but exhibited inhibitory effects on apoptosis (Figures S4A,B and S5A,B, Supporting Information). These findings are consistent with the results of cell viability assays. Furthermore, overexpression of EBBP attenuated pro‐cell death effects induced by erastin, a well‐characterized ferroptosis activator, whereas EBBP knockdown significantly exacerbated this effect (Figure S3C,D, Supporting Information). Additionally, correlation analysis using the GTEx database revealed a positive association between EBBP expression and anti‐ferroptosis genes (GPX4, SLC7A11, SLC3A2, FTH1, GCLM, GCLC) in human left ventricular specimens. In contrast, no significant correlation was observed with inflammatory markers like NFKBIA, CXCL1, or CXCL2 (Figure S3E,F, Supporting Information). Collectively, these results suggest that EBBP may predominantly protect against doxorubicin‐induced cardiomyopathy by inhibiting ferroptosis.

In order to validate the aforementioned findings, we assessed the impact of EBBP on ferroptosis in DOX‐challenged cardiomyocytes. Ferroptosis is defined by lipid peroxidation, excess iron accumulation, and intracellular reactive oxygen species (ROS) accumulation. We found that EBBP overexpression markedly suppressed the elevated expression of the ferroptosis marker *Ptgs2* (Figure S6A, Supporting Information), inhibited lipid peroxide generation as assessed by flow cytometry employing C11‐BODIPY (Figure S6B, Supporting Information), and reduced malondialdehyde (MDA) levels induced by DOX in H9c2 cells (Figure S6C, Supporting Information). Furthermore, EBBP overexpression mitigated DOX‐induced ROS accumulation (Figure S6D,E, Supporting Information), prevented iron overload (Figure S6F,G,J, Supporting Information), and ameliorated mitochondrial dysfunction, as evidenced by a significant increase in mitochondrial membrane potential (Figure S6H,I, Supporting Information).

To further validate the necessity of EBBP in ferroptosis suppression, we performed loss‐of‐function experiments. Knockdown of EBBP exacerbated DOX‐induced ferroptotic responses, elevating *Ptgs2* mRNA expression (Figure 4D), lipid peroxidation (Figure 4E), MDA levels (Figure 4F), ROS accumulation (Figure 4G,J), and intracellular iron levels (Figure 4H,K; Figure S6K, Supporting Information), while aggravating mitochondrial dysfunction (Figure 4I,L). Critically, these effects were largely rescued by the lipid peroxide scavenger, Fer‐1, except for iron accumulation (Figure 4D–J). Taken together, EBBP inhibits anthracycline‐induced cardiomyocyte ferroptosis in vitro.

### 2.6 EBBP Attenuates Anthracycline‐Induced Myocardial Ferroptosis In Vivo

**Figure 5:**
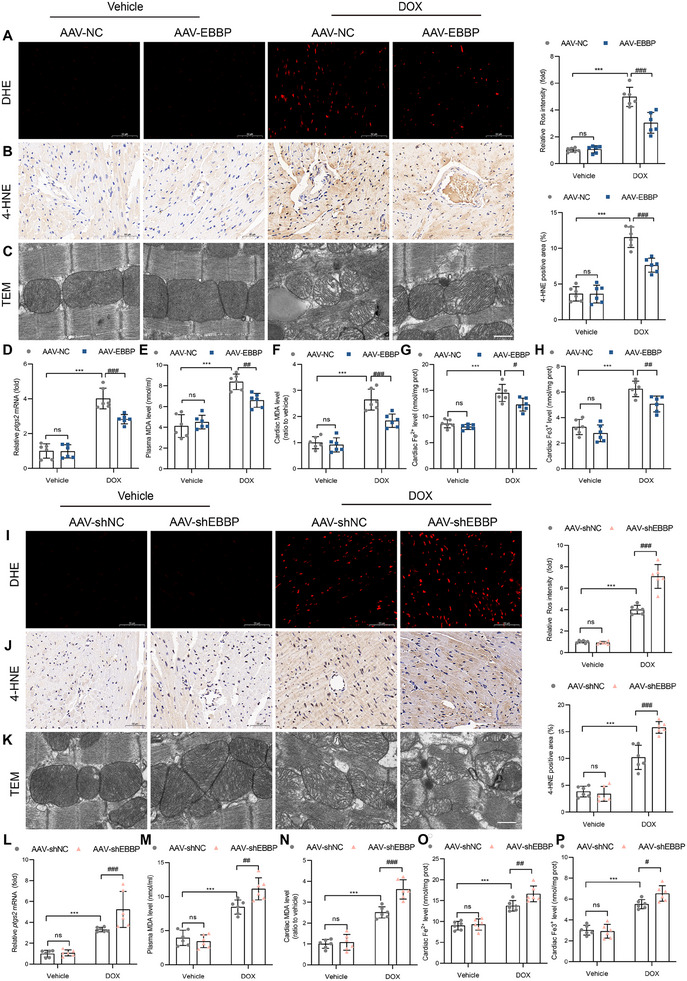
EBBP attenuates anthracycline‐induced myocardial ferroptosis in vivo. A–H) AAV‐NC or AAV‐EBBP was administered via tail vein injection, and the DoIC model was established 14 days thereafter. A) Representative images and quantification of fluorescent immunohistochemistry staining for DHE in mouse myocardium (scale bars, 100 µm, *n* = 6). B) Representative images and quantification of immunohistochemistry for 4‐HNE in mouse myocardium (scale bars, 50 µm, *n* = 6). C) Representative transmission electron micrographs of cardiac tissues (Scale bar, 500 nm). D) The mRNA levels of Ptgs2 mRNA in cardiac tissues (*n* = 6). E) Plasma MDA level (*n* = 6). F) MDA levels in cardiac tissues (*n* = 6). G,H) Fe^2+^ and Fe^3+^ levels in cardiac tissues (*n* = 6). I–P) AAV‐shNC or AAV‐shEBBP was administered via tail vein injection, and the DoIC model was established 14 days thereafter. I) Representative images and quantification of fluorescent immunohistochemistry staining for DHE in mouse myocardium (scale bars, 100 µm, *n* = 6). J) Representative images and quantification of immunohistochemistry for 4‐HNE in mouse myocardium (scale bars, 50 µm, *n* = 6). K) Representative transmission electron micrographs of cardiac tissues (Scale bar, 500 nm). L) The mRNA levels of Ptgs2 mRNA in cardiac tissues (*n* = 6). M) Plasma MDA level (*n* = 6). N) MDA levels in cardiac tissues (*n* = 6). O‐P) Fe^2+^ and Fe^3+^ levels in cardiac tissues (*n* = 6). Values are presented as the mean ± SD. ***p < 0.001 vs Vehicle + AAV‐NC or Vehicle + AAV‐shNC; ^#^ *p* < 0.05, ^##^ *p* < 0.01 and ^###^ *p* < 0.001 vs DOX + AAV‐NC or DOX + AAV‐ shNC group.

To further verify the critical role of EBBP in anthracycline‐induced myocardial ferroptosis, ferroptosis‐associated parameters were systematically analyzed through in vivo models. Specifically, we quantified the levels of ROS and 4‐hydroxynonenal (4‐HNE), a marker of lipid peroxidation and ferroptosis.^[^ ^16^, ^37^ ^]^ The results demonstrated that DOX administration significantly increased ROS and 4‐HNE levels in the heart. Overexpression of EBBP efficiently attenuated the elevation of ROS and 4‐HNE (**Figure** **5**A,B), whereas EBBP knockdown markedly exacerbated this elevation (Figure 5I,J). Transmission electron microscopy (TEM) analysis revealed that EBBP overexpression alleviated DOX‐induced mitochondrial dysfunction, characterized by mitochondrial swelling, vacuolization, and disintegration/lysis of cristae (Figure 5C), whereas EBBP deficiency amplified these ultrastructural abnormalities (Figure 5K).

Furthermore, DOX treatment resulted in upregulation of cardiac *Ptgs2* mRNA in WT mice. This upregulation was significantly suppressed by EBBP overexpression (Figure 5D) but exacerbated by EBBP knockdown (Figure 5L). In addition, the levels of MDA and iron were examined. Our findings indicated that DOX‐induced increases in serum MDA levels (Figure 5E), cardiac MDA levels (Figure 5F), and iron levels (Figure 5G,H) were alleviated by EBBP overexpression but further exacerbated by its knockdown (Figure 5M–P). Collectively, these data demonstrate that EBBP protects against anthracycline‐induced myocardial ferroptosis.

### 2.7 EBBP Inhibits Anthracycline‐Induced Cardiomyocyte Ferroptosis by Regulating SLC7A11/GSH/GPX4 and Iron Homeostasis

Subsequently, we explored the cytoprotective mechanism of EBBP against ferroptosis induced by anthracycline. Ferroptosis results from an imbalance between iron‐dependent lipid peroxidation and antioxidant mechanisms that suppress lipid peroxidation. On the one hand, our prior findings demonstrated that EBBP can mitigate iron overload in DOX‐treated mice and cardiomyocytes. Cellular iron homeostasis is dynamically maintained through three key components: 1) TfR‐mediated iron import, 2) FPN‐mediated iron export, and 3) ferritin nanocage storage via FTH/FTL heteropolymers.^[^ ^38^ ^]^ On the other hand, the transcriptomic profiling revealed that EBBP could significantly upregulate the expression of SLC7A11. The SLC7A11/GSH/GPX4 axis represents a central defense pathway for suppressing ferroptosis by eliminating lipid peroxides.^[^ ^39^ ^]^ SLC7A11 (also known as xCT), a cystine/glutamate antiporter, mediates extracellular cystine uptake through glutamate‐coupled exchange. Cystine is reduced to cysteine, serving as a precursor for GSH synthesis. GPX4 subsequently utilizes GSH as a cofactor to reduce lipid peroxides, thereby inhibiting ferroptosis. These findings prompted us to investigate how EBBP regulates iron homeostasis and its relationship with the SLC7A11/GSH/GPX4 pathway.

**Figure 6:**
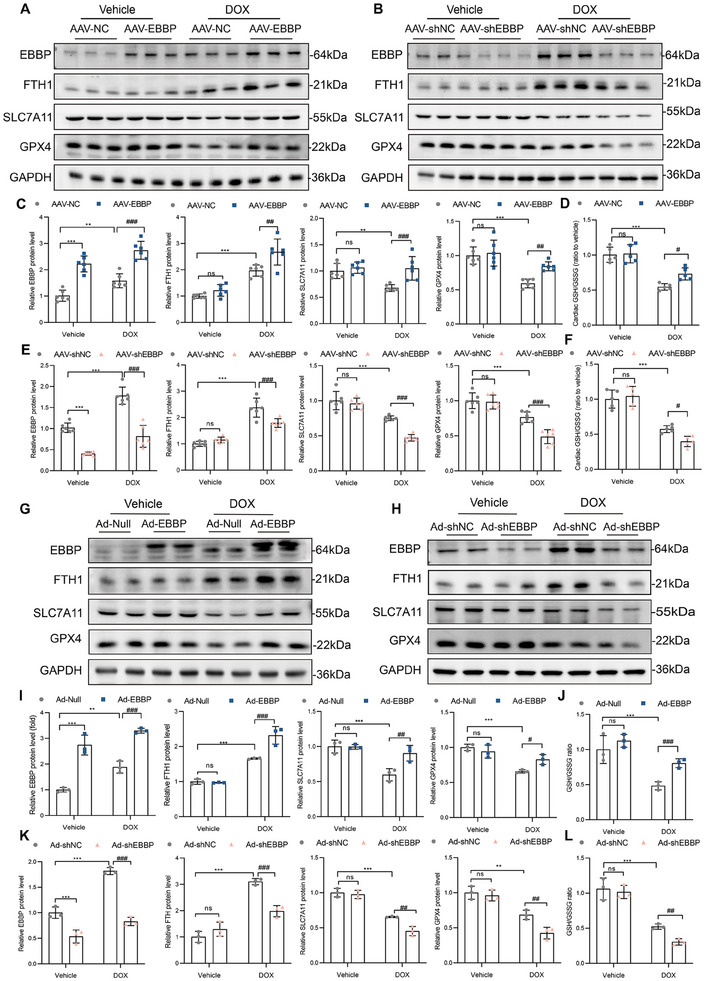
EBBP inhibits anthracycline‐induced cardiomyocyte ferroptosis by regulating SLC7A11/GSH/GPX4 and iron homeostasis. A,B) Immunoblots for EBBP, FTH1, SLC7A11, and GPX4 in cardiac tissues. C,D) Statistical analysis of the protein level of EBBP, FTH1, SLC7A11, GPX4, and GSH/GSSG in cardiac tissues administered with AAV‐NC or AAV‐EBBP (*n* = 6). E,F) Statistical analysis of the protein level of EBBP, FTH1, SLC7A11, GPX4, and GSH/GSSG in cardiac tissues administered with AAV‐shNC or AAV‐shEBBP (*n* = 6). G,H) Representative western blots of EBBP, FTH1, SLC7A11, and GPX4 in H9c2 cells. I,J) Statistical analysis of the protein level of EBBP, FTH1, SLC7A11, GPX4, and GSH/GSSG in H9c2 cells infection with Ad‐Null or Ad‐EBBP (*n* = 3). K,L) Statistical analysis of the protein level of EBBP, FTH1, SLC7A11, GPX4, and GSH/GSSG in H9c2 cells infection with Ad‐shNC or Ad‐shEBBP (*n* = 3). Values are presented as the mean ± SD. *p < 0.05, **p < 0.01 and ***p < 0.001 vs Vehicle + Ad‐Null or Vehicle+ Ad‐shNC; ^#^ *p* < 0.05, ^##^ *p* < 0.01 and ^###^ *p* < 0.001 vs DOX+ Ad‐Null or DOX+ Ad‐shNC.

In vivo, our results demonstrated that EBBP overexpression effectively reversed the DOX‐induced downregulation of cardiac SLC7A11 and GPX4 expression and reduction in the GSH/GSSG ratio. Moreover, EBBP overexpression also significantly enhanced the expression of FTH1 (**Figure** **6**A,C,D). In contrast, cardiac knockdown of EBBP exacerbated DOX‐induced reduction of SLC7A11, GPX4, and the GSH/GSSG. Furthermore, EBBP knockdown also reduced the expression of FTH1 under DOX treatment (Figure 6B,E,F).

Consistent with in vivo findings, overexpression of EBBP resulted in elevated expression of SLC7A11, GPX4, and FTH1, as confirmed by immunoblotting, and partially reinstated the GSH/GSSG ratio in H9c2 cells (Figure 6G,I,J). Nonetheless, the depletion of EBBP markedly reduced the expression of SLC7A11, GPX4, and FTH1 while further exacerbating the reduction of cellular GSH/GSSG (Figure 6H,K,L). Importantly, EBBP did not alter expression of pro‐ferroptotic regulators ACSL4, ALOX15, TFR1 (Figure S7A, Supporting Information), indicating that EBBP mitigates anthracycline cardiotoxicity mainly by modulation of SLC7A11/GSH/GPX4 axis and iron homeostasis.

### 2.8 EBBP Activates Nrf2 and ATF4 Through PERK‐Mediated ISR

To further explore the mechanism by which EBBP regulates the expression of SLC7A11/GPX4 and FTH1, the mRNA levels of these genes were quantitatively analyzed. DOX stimulation increased the mRNA levels of SLC7A11/GPX4 and FTH1 (Figure S7B,C, Supporting Information). Surprisingly, the changes in mRNA and protein levels of SLC7A11/GPX4 were inconsistent under DOX treatment, which may be attributed to additional regulation of protein levels by translational control and/or protein stability mechanisms.^[^ ^40^, ^41^ ^]^ Despite this discrepancy, our findings confirmed that EBBP transcriptionally upregulated the expression of SLC7A11/GPX4 and FTH1.

Subsequently, we extracted differentially expressed genes (DEGs) from the GSE233644 dataset and performed transcription factor enrichment analysis using the ChEA3 database (ChIP‐X Enrichment Analysis Version 3) to identify key regulators implicated in doxorubicin‐induced cardiomyopathy. The most enriched transcription factors among the top candidates included ATF3, NME2, ATF4, Nrf2, ZNF398, ZNF706, ZNF581, SOX4, YBX1, and p53. We then utilized the GTRD database to predict transcription factors regulating the transcriptional activity of SLC7A11/GPX4 and FTH1. By performing an intersection analysis, we identified four transcription factors (ATF3, ATF4, Nrf2, and p53) that may regulate the expression of SLC7A11/GPX4 and FTH1 and play critical roles in anthracycline‐induced cardiomyopathy (Figure S8A, Supporting Information).

**Figure 7:**
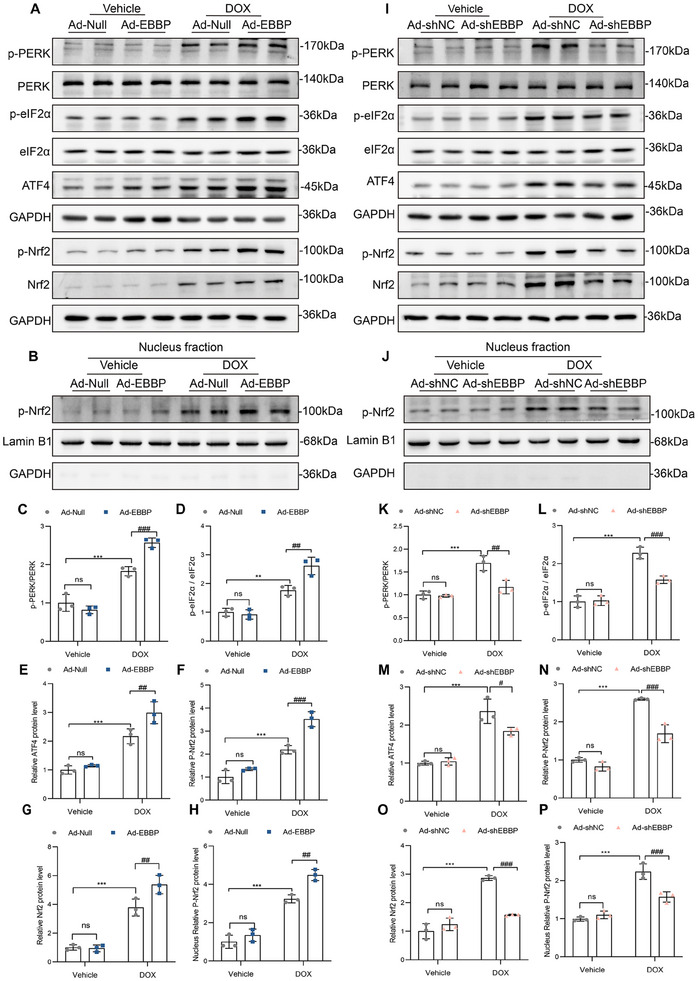
EBBP promotes the activation of the PERK/ATF4 axis and Nrf2. A) Following infection with Ad‐Null or Ad‐EBBP, H9c2 was subjected to DOX (1 µm) for 24 h. Representative western blots of p‐PERK, PERK, p‐ eIF2α, eIF2α, ATF4, p‐Nrf2, and Nrf2 in H9c2 cells. B) Representative western blots of nuclear p‐Nrf2 in H9c2 cells. C–H) Statistical analysis of the protein levels in (A,B) (*n* = 3). I) Following infection with Ad‐shNC or Ad‐shEBBP, H9c2 was subjected to DOX (1 µm) for 24 h. Representative western blots of p‐PERK, PERK, p‐eIF2α, eIF2α, ATF4, p‐Nrf2, and Nrf2 in H9c2 cells. J) Representative western blots of nuclear p‐Nrf2 in H9c2 cells. K–P) Statistical analysis of the protein levels in (I,J) (*n* = 3). Values are presented as the mean ± SD. **p < 0.01 and ***p < 0.001 vs Vehicle + Ad‐Null or Vehicle+ Ad‐shNC; ^#^ *p* < 0.05, ^##^ *p* < 0.01 and ^###^ *p* < 0.001 vs DOX+ Ad‐Null or DOX+ Ad‐shNC.

We found that EBBP did not affect the expression of p53 and ATF3 (Figure S8B, Supporting Information). However, EBBP overexpression upregulated ATF4 expression and activated Nrf2, as evidenced by elevated levels of phosphorylated Nrf2 (**Figure** **7**A,E–G). Conversely, EBBP knockdown suppressed both ATF4 expression and Nrf2 activation (Figure 7I,M–O). Nrf2 plays a pivotal function in alleviating ferroptosis by regulating several essential genes involved in iron metabolism and antioxidant defense pathways. Under homeostatic settings, Nrf2 undergoes ubiquitylation and subsequent proteasomal degradation. However, in response to stress such as DOX stimulation, Nrf2 translocates in the nucleus to initiate transcription of target genes.^[^ ^42^ ^]^ Our results confirmed that EBBP overexpression promoted the nuclear translocation of Nrf2 under DOX treatment (Figure 7B,H), whereas EBBP depletion markedly inhibited this process (Figure 7J,P). ATF4 serves as a central mediator of the integrated stress response (ISR), orchestrating anti‐ferroptosis mechanisms.^[^ ^26^ ^]^ Subsequent analysis revealed that EBBP overexpression enhanced transcriptional activation of Nrf2‐regulated genes (e.g., *Gclm*, *Gclc*, *Ho1*, *Nqo1*) and ATF4‐target genes (e.g., *Cth*, *Psph*, *Slc1a4*) (Figure S8C,E, Supporting Information), with opposite effects observed upon EBBP knockdown (Figure S8D,F, Supporting Information).

The PERK/eIF2α axis constitutes a pivotal component of the ISR,^[^ ^25^ ^]^ coordinating ATF4 activation and Nrf2 phosphorylation to facilitate nuclear translocation.^[^ ^43^ ^]^ Furthermore, recent studies have demonstrated that the PERK branch protects the heart against ischemia/reperfusion injury and pressure overload‐induced hypertrophy.^[^ ^25^ ^]^ However, it remains unclear whether the PERK/eIF2α branch of the ISR is involved in DOX‐induced cardiotoxicity. Consequently, we investigated the expression levels of the PERK/eIF2α axis. Our analysis revealed that DOX treatment significantly increased phosphorylation levels of both PERK and its downstream effector protein, eIF2α. Although EBBP overexpression did not alter total PERK expression, it potentiated DOX‐induced phosphorylation of PERK and eIF2α (Figure 7A,C,D). Conversely, EBBP silencing reduced the phosphorylation of PERK and eIF2α (Figure 7I,K,L).

To determine whether EBBP‐mediated Nrf2 activation requires PERK/eIF2α activation, we employed pharmacological inhibition using GSK2606414. Treatment with this PERK‐specific inhibitor completely blocked EBBP‐induced activation of the PERK/eIF2α/ATF4 axis in DOX‐treated H9c2 cells (Figure S9A, Supporting Information). Notably, GSK2606414 treatment also abolished both the EBBP‐mediated increase in Nrf2 phosphorylation and the subsequent nuclear translocation of Nrf2 under DOX stimulation (Figure S9B, Supporting Information).

### 2.9 EBBP Attenuates Anthracycline‐Induced Cardiomyocyte Ferroptosis by PERK‐Mediated ISR

Subsequently, we investigated the role of PERK in the EBBP‐mediated ferroptosis regulation in DOX‐treated cardiomyocytes. Comparative analysis revealed that pharmacological inhibition of PERK in EBBP‐overexpressing cells (DOX+ad‐EBBP+PERK inhibitor) significantly attenuated the cardioprotective effects observed in DOX+ad‐EBBP treated cells. Specifically, PERK inhibition resulted in markedly decreased cell viability (Figure S10A, Supporting Information) accompanied by substantial increases in multiple ferroptosis markers, including *Ptgs2* mRNA expression (Figure S10B, Supporting Information), lipid peroxide accumulation (Figure S10C, Supporting Information), MDA levels (Figure S10D, Supporting Information), ROS production (Figure S10E,H, Supporting Information) and intracellular Fe^2+^ content (Figure S10F,I, Supporting Information). The PERK inhibitor also entirely abolished the protective effect of EBBP on mitochondrial membrane potential under DOX stimulation (Figure S10G,J, Supporting Information). Importantly, in DOX‐treated H9c2 cells, administration of the PERK inhibitor GSK2606414 significantly suppressed the EBBP‐induced upregulation of key anti‐ferroptotic proteins (SLC7A11, GPX4, and FTH1) and prevented the improvement in cellular redox status as measured by GSH/GSSG ratio (Figure S10K,L, Supporting Information).

These findings collectively demonstrate that PERK activation is essential for EBBP‐mediated protection against DOX‐induced cardiomyocyte ferroptosis.

### 2.10 Pharmacological Inhibition of PERK Attenuates EBBP's Cardioprotective Effects in Anthracycline‐Induced Cardiotoxicity

**Figure 8:**
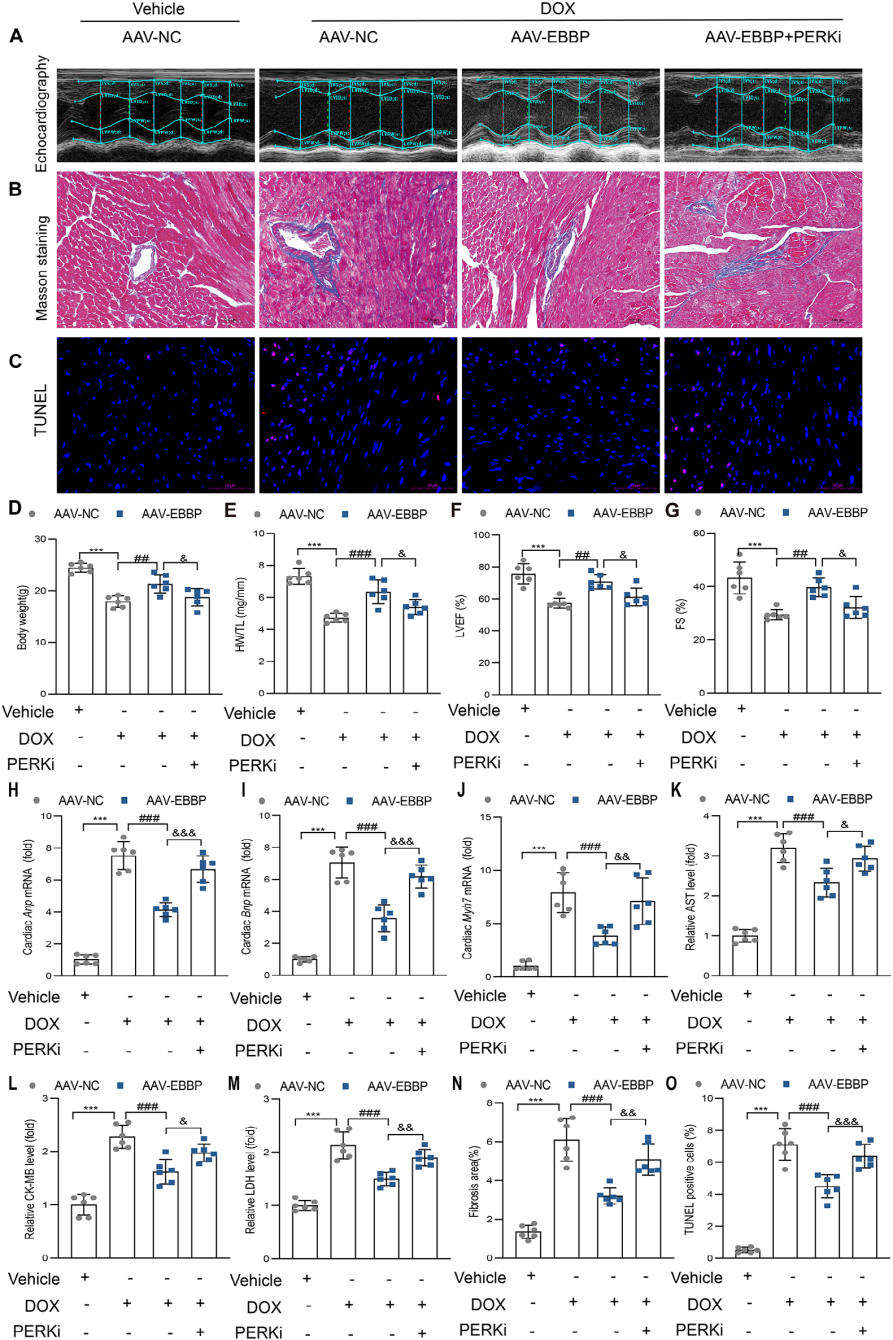
Pharmacological inhibition of PERK attenuates EBBP's cardioprotective effects in anthracycline‐induced cardiac injury. A) Images of transthoracic echocardiography. B) Representative images of Masson's trichrome staining in heart (scale bars, 100 µm). C) Representative images of TUNEL staining in heart (scale bars, 50 µm). D) Changes in body weight of mice after DOX injection (*n* = 6). E) Changes in HW/TL (*n* = 6). F‐G) Quantification analysis of LVEF and FS (*n* = 6). H–J) The mRNA levels of Anp, Bnp, and Myh7 in cardiac tissues (*n* = 6). K–M) Quantitative analysis of serum AST, CK‐MB, and LDH levels (*n* = 6). N) Statistical analysis of fibrosis area (*n* = 6). O) Quantitative analysis of TUNEL‐positive cells via immunofluorescence staining (*n* = 6). Values are presented as the mean ± SD. ***p < 0.001 vs Vehicle + AAV‐NC; ^##^ *p* < 0.01 and ^###^ *p* < 0.001 vs DOX + AAV‐NC group; ^&&&^ *p* < 0.001 vs DOX + AAV‐EBBP group.

To establish whether EBBP's cardioprotective effects against anthracycline‐induced cardiac dysfunction are PERK‐dependent in vivo, we administered GSK2606414 (a selective PERK inhibitor) to mice before DOX exposure. Cardiac‐specific EBBP overexpression demonstrated significant improvements in body weight and HW/TL (**Figure** **8**D,E), along with enhanced cardiac function (Figure 8A,F–M). Histological analysis revealed preserved myofibrillar architecture (Figure S11A), reduced cardiac collagen deposition (Figure 8B,N), and diminished DNA damage (Figure 8C,O). However, these protective effects were markedly attenuated by PERK inhibition.

Moreover, PERK inhibition also blunted the protective effects of EBBP against anthracycline‐triggered myocardial ferroptosis. The level of ROS (Figure S11B,E, Supporting Information), 4‐HNE (Figure S11C,F, Supporting Information), cardiac *Ptgs2* mRNA, serum and cardiac MDA (Figure S11G–I, Supporting Information), and cardiac iron (Figure S11K,L, Supporting Information) were higher in AAV‐EBBP+PERKi+DOX mice compared to AAV‐EBBP+DOX mice. Furthermore, PERK inhibition reversed EBBP‐induced GSH elevation (Figure S11J, Supporting Information) and exacerbated mitochondrial ultrastructural damage (Figure S11D, Supporting Information).

These findings collectively establish that EBBP mitigates anthracycline‐induced cardiomyopathy through PERK‐mediated integrated stress response activation.

### 2.11 EBBP Promotes PERK Activation Through the Non‐Degradative Ubiquitination of GRP78

**Figure 9:**
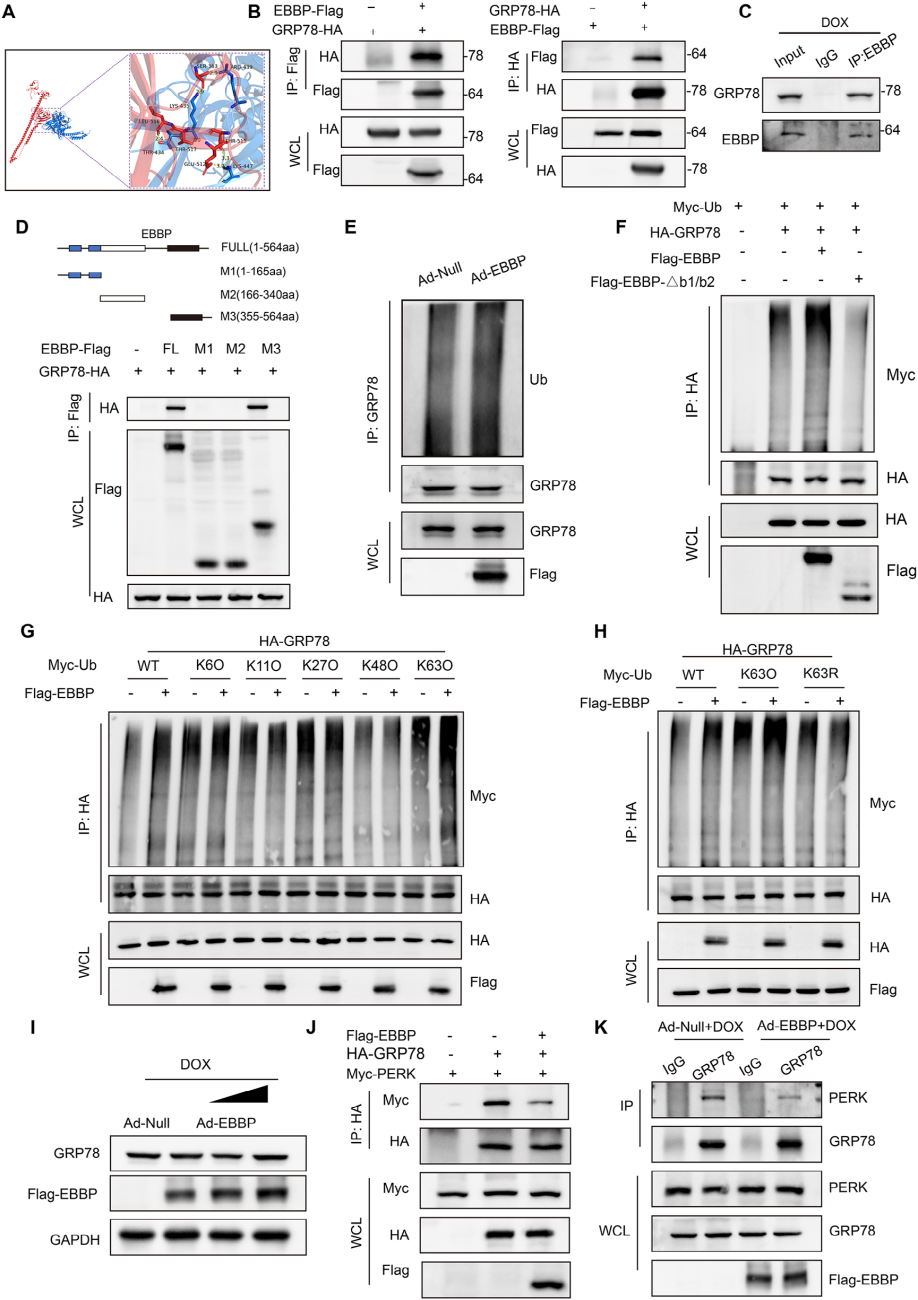
EBBP promotes PERK activation through the non‐degradative ubiquitination of GRP78. A) Molecular docking predicted an interaction between EBBP and GRP78. Red, EBBP. Blue, GRP78. B) Coimmunoprecipitation (co‐IP) experiments of the interaction between EBBP and GRP78 in 293T cells. WCL, whole‐cell lysis. C) In DOX‐treated NRCMs, an endogenous IP study was performed to investigate the interaction between EBBP and GRP78. D) A diagrammatic representation of the domains of EBBP and the shortened mutants is shown. Co‐IP analysis of the interaction domains of EBBP and GRP78. E) NRCMs that have been transfected with Ad‐Null or Ad‐EBBP were subjected to ubiquitination tests in order to determine the level of ubiquitination of endogenous GRP78. F) Ubiquitination assays determine the ubiquitination of HA‐GRP78 in HEK293T cells. G,H) The ubiquitination of HA‐GRP78 in response to Flag‐EBBP transfection was investigated in HEK293T cells transfected with the wild‐type (WT) and mutant Myc‐Ub plasmids to determine possible lysine ubiquitination types. I) Representative western blots of GRP78 and EBBP in NRCMs infected with Ad‐EBBP followed by stimulation with DOX (1 um for 24 h). J) Assays of coimmunoprecipitation (co‐IP) between GRP78 and PERK in 293T cells. K) Endogenous IP analysis of the interaction between GRP78 and PERK in NRCMs treated with DOX was investigated, aiming to determine how EBBP overexpression influences this interaction.

Next, we explored the molecular mechanisms underlying the EBBP‐mediated PERK activation. As EBBP functions as an E3 ligase, we initially assessed whether EBBP interacted with PERK directly. However, co‐immunoprecipitation (Co‐IP) assays demonstrated no direct binding between EBBP and PERK (Figure S12A,B, Supporting Information), suggesting an indirect regulatory mechanism. We then focused on glucose‐regulated protein 78 (GRP78), the canonical PERK chaperone that maintains its inactive state through physical association under physiological conditions. However, under stress conditions, GRP78 dissociates from PERK, thereby activating PERK signaling to adapt to the stress response. Molecular docking analysis revealed a potential interaction interface between EBBP and GRP78 (**Figure** **9**A). We further validated the interaction between EBBP and GRP78 through exogenous immunoprecipitation experiments in 239T (Figure 9B) and endogenous immunoprecipitation assays performed in DOX‐treated NRCMs (Figure 9C). Concerning the stress‐dependent regulation of EBBP‐mediated PERK activation, we hypothesize that distinct interaction patterns between EBBP and GRP78 may emerge under pathological conditions compared to physiological states. Notably, our experiments demonstrated that EBBP directly interacted with GRP78, and this molecular interaction exhibiting significant augmentation in cardiomyocytes following DOX exposure (Figure S12C, Supporting Information). Subsequently, we identified the specific domains that were accountable for protein interaction. To this end, we generated truncated mutants of both EBBP and GRP78 for coimmunoprecipitation experiments. Results showed that the SPRY region (355‐564aa) of EBBP was responsible for interacting with the SBD domain of GRP78 (Figure 9D; Figure S12D, Supporting Information).

We next explored the impact of EBBP on the ubiquitination state of GRP78. EBBP promoted the ubiquitination of GRP78 in both endogenous and exogenous assays (Figure 9E,F). Our results also indicated that the B1/B2 domains, previously identified as crucial for the E3 ligase activity of EBBP,^[^ ^44^, ^45^ ^]^ significantly contributed to the ubiquitination of GRP78, as their deletion markedly diminished GRP78 ubiquitination (Figure 9F). Then, we performed ubiquitination assays employing mutant variants of ubiquitin. EBBP specifically catalyzed GRP78 modification with WT ubiquitin and K63O (Ub with the intact Lysine63 residing alone), but not K6O, K11O, K27O, or K48O (Figure 9G). Moreover, EBBP failed to catalyze the linkage of K63R (Ub only Lysine63 residing was mutated) to GRP78 (Figure 9H). Collectively, these findings indicate that EBBP predominantly mediates the K63‐linked ubiquitination of GRP78.

Then, we further explored the effect of EBBP‐mediated ubiquitination on GRP78. Notably, western blot analysis revealed that total GRP78 protein levels remained unchanged in cardiomyocytes overexpressing EBBP (Figure 9I). Moreover, the protein turnover of GRP78 remained unaffected by EBBP overexpression (Figure S12E, Supporting Information). Interestingly, we found that EBBP reduced the amount of the PERK that coimmunoprecipitated with GRP78 in 293T (Figure 9J). However, deletion of the B1/B2 domains of EBBP, which are crucial for the E3 ligase activity of EBBP, abolished this effect (Figure S12F, Supporting Information), indicating that EBBP's ability to modulate the interaction between PERK and GRP78 is dependent on its ubiquitin ligase function. In cardiomyocytes exposed to doxorubicin, we further observed that overexpression of EBBP attenuated the interaction between PERK and GRP78 (Figure 9K), whereas knockdown of EBBP reversed the DOX‐induced dissociation of PERK from GRP78 (Figure S12G, Supporting Information). Collectively, these findings demonstrate that EBBP directly binds to GRP78 and facilitates its ubiquitination by K63 linkage, thereby reducing the repressive interaction between GRP78 and PERK, resulting in the phosphorylation of PERK.

### 2.12 Fer‐1 Abrogates the Detrimental Effect of EBBP Deficiency on Anthracycline‐Induced Cardiotoxicity

Finally, to confirm that the protective effect of EBBP against anthracycline‐induced cardiomyopathy is mediated through the inhibition of ferroptosis in vivo, mice received daily intraperitoneal injections of Fer‐1, a selective inhibitor of ferroptosis during the induction of DoIC. Our results demonstrated that EBBP knockdown exacerbated DOX‐induced body weight loss and cardiac atrophy, as evidenced by reduced body weight and HW/TL ratios (Figure S13D,E, Supporting Information). This intervention also resulted in impaired cardiac function (Figure S13A,F–J, Supporting Information), pronounced myocardial injury (Figure S13B,K–N, Supporting Information), and elevated ROS accumulation accompanied by enhanced lipid peroxidation (Figure S13C,O,P, Supporting Information). Consistent with previous studies, we observed that Fer‐1 exerted a protective effect against DOX‐induced cardiac injury and ferroptosis. Notably, Fer‐1 treatment effectively rescued the above detrimental effects of EBBP deficiency.

## 3 Discussion

Anthracyclines, including doxorubicin, daunorubicin, epirubicin, and idarubicin, represent indispensable chemotherapeutic agents for treating various cancers.^[^ ^5^ ^]^ However, their clinical application is limited by dose‐dependent cardiotoxicity, which poses significant health risks and may compromise patient survival. Currently, no effective pharmacological interventions are available to prevent chemotherapy‐induced cardiotoxicity. Therefore, identifying potential therapeutic targets to mitigate anthracycline‐associated cardiac damage is critically important.

Through systematic analysis of transcriptomic profiles from doxorubicin‐induced cardiomyopathy models (GEO accession numbers: GSE40289, GSE226116, GSE233644), we identified 11 candidate genes potentially implicated in disease pathogenesis. Subsequent validation experiments revealed that EBBP exhibited the most pronounced differential expression in DOX‐treated hearts compared to controls. Functional studies demonstrated that cardiac‐specific EBBP overexpression alleviated DOX‐induced cardiomyopathy, while EBBP knockdown exacerbated cardiac damage through modulation of ferroptosis. Notably, we discovered a novel mechanism whereby EBBP promotes non‐degradative ubiquitination of GRP78, disrupting its inhibitory interaction with PERK and activating the PERK‐mediated ISR. This signaling cascade ultimately activates downstream effectors ATF4 and Nrf2, which transcriptionally upregulate the SLC7A11/GSH/GPX4 antioxidant axis while restoring iron homeostasis. These findings position EBBP as a promising therapeutic target for preventing anthracycline‐induced cardiotoxicity.

Multiple mechanisms are involved in regulating anthracycline‐induced cardiotoxicity, such as DNA damage, disruption of autophagy, oxidative stress, and various forms of cell death.^[^ ^15^ ^]^ Previous studies indicated that EBBP exerted protective effects against oxidative damage and inflammation in nonalcoholic steatohepatitis.^[^ ^30^ ^]^ Chauhan et al. demonstrated that EBBP confers cellular protection against lysosomal damage through modulation of selective autophagy.^[^ ^29^ ^]^ EBBP also enhances cellular stress resistance via activation of the p62‐Nrf2 signaling axis.^[^ ^45^ ^]^ Furthermore, EBBP inhibited pathological cardiac hypertrophy and ischemia‐reperfusion injury.^[^ ^31^, ^32^ ^]^ More importantly, some investigations have identified EBBP as a tumor suppressor in various malignancies.^[^ ^46^, ^47^ ^]^ In this regard, we are further convinced that EBBP may play a critical role in regulating of anthracycline‐induced cardiotoxicity. In our research, EBBP overexpression notably mitigated myocardial injury and inflammation and improved cardiac function, while the knockdown of EBBP exhibited opposite effects. Given that previous studies have reported EBBP's ability to promote the secretion of IL‐1β in COS‐1 cells,^[^ ^48^ ^]^ we also investigated the levels of inflammatory factors in the supernatant of cardiomyocytes exposed to doxorubicin (DOX). The results demonstrated that EBBP exerted no significant impact on the levels of inflammatory cytokine (IL‐1β, IL‐6, TNF‐α) in the supernatant (Figure S2H,I). This apparent discrepancy may stem from EBBP's cell type‐specific functionality and context‐dependent regulatory mechanisms under distinct pathological conditions. The observed anti‐inflammatory effects in vivo could potentially result from EBBP‐mediated mitigation of cardiac tissue damage, thereby reducing inflammatory cell infiltration indirectly. The precise molecular basis underlying these tissue‐specific manifestations requires further elucidation through targeted mechanistic studies.

Recently, several studies have employed a variety of cell death inhibitors to investigate the pathogenesis of doxorubicin‐induced cardiomyopathy. Murine model studies have confirmed that ferroptosis inhibitor Fer‐1 effectively attenuates DOX‐induced cardiac dysfunction. Notably, caspase inhibitor emricasan and necroptosis inhibitor necrostatin‐1 failed to improve murine survival outcomes.^[^ ^16^ ^]^ Complementary in vitro investigations by Wang et al. demonstrated that ferroptosis inhibitors (Fer‐1 and deferoxamine mesylate) significantly ameliorated DOX‐induced cytotoxicity in H9c2 cardiomyocytes, whereas necroptosis and autophagy modulators showed minimal efficacy.^[^ ^49^ ^]^ Our experimental data revealed that autophagy inhibitor 3‐MA, autophagy inducer rapamycin, and necroptosis inhibitor Nec‐1 exhibited no cytoprotective effects against DOX‐induced cell death. Conversely, ferroptosis inhibitor Fer‐1, iron chelator DFO, apoptosis inhibitor ZVAD‐FMK, and pyroptosis inhibitor belnacasan demonstrated significant protection, with Fer‐1 and DFO showing superior efficacy. These findings corroborate previous reports,^[^ ^16^, ^36^ ^]^ suggesting ferroptosis predominance in DOX‐mediated cytotoxicity. Intriguingly, current and prior investigations consistently demonstrate the ineffectiveness of autophagy modulators in preventing DOX‐induced cardiomyocyte death. This phenomenon likely stems from the dual regulatory nature of autophagy in DOX cardiotoxicity. Doxorubicin exerts dual regulatory effects on autophagy, with both enhancing and inhibitory capacities. Furthermore, its impact on autophagic activity demonstrates dose‐dependent variations.^[^ ^50^ ^]^ These complexities preclude the elucidation of autophagy's role in doxorubicin‐induced cardiomyopathy through isolated application of autophagic modulators in vitro experimental models. Therefore, we conducted a comprehensive investigation into the effects of EBBP on autophagic flux regulators. The results demonstrated that EBBP did not exert any significant impact on the protein levels of LC3‐II, P62, or Atg7, nor did it modulate the expression of PARKIN and PINK1, which are established regulators of mitophagy. Similarly, pyroptosis and necroptosis pathway components remained unaffected by EBBP modulation. However, our results demonstrate that ferroptosis inhibitors (Fer‐1 and DFO) most effectively suppress the exacerbating effect of EBBP knockdown on DOX‐induced cell death. KEGG enrichment analysis of transcriptomic data further confirmed EBBP's capacity to modulate ferroptosis‐associated pathways. Thus, these findings consistently demonstrate that EBBP mitigates DOX‐induced cardiotoxicity primarily by suppressing ferroptosis.

Ferroptosis is a kind of cell death that is caused by iron‐dependent lipid peroxidation. Various metabolic pathways modulate ferroptosis, such as redox homeostasis, iron, amino acids, and lipids metabolism.^[^ ^11^ ^]^ The SLC7A11/GSH/GPX4 axis constitutes a canonical anti‐ferroptotic mechanism, wherein system Xc− (comprising SLC7A11 and SLC3A2 subunits) mediates cystine import, subsequently reduced to cysteine for glutathione (GSH) biosynthesis. GPX4 can facilitate the reduction of phospholipid hydroperoxides (PLOOHs) to their corresponding alcohols (PLOHs), thus protecting cells against ferroptosis.^[^ ^39^ ^]^ In addition, lipid peroxidation is dependent on the Fenton reaction, which is facilitated by Fe^2+^ and iron‐dependent enzymes, including arachidonate lipoxygenases (ALOXs). The maintenance of iron homeostasis involves the modulation of the TFR, ferritin, and FPN. DOX could cause iron overload, resulting in cardiomyocyte ferroptosis.^[^ ^51^ ^]^ Recently, Pan et al. also revealed that DOX‐challenged cardiomyocytes exhibit impaired iron homeostasis, and treatment with deferoxamine or dexrazoxane inhibited myocardial ferroptosis, thereby mitigating doxorubicin‐induced cardiotoxicity.^[^ ^38^ ^]^ Moreover, deletion of ferritin H in mice promotes cardiomyopathy ferroptosis.^[^ ^52^ ^]^ Our experimental data revealed that EBBP overexpression confers cardio‐protection against anthracycline‐induced cardiomyocyte ferroptosis via coordinated upregulation of the SLC7A11/GPX4 axis, FTH1 expression and enhanced GSH/GSSG redox balance in both murine models and cardiomyocytes. Conversely, EBBP knockdown exacerbated these effects. These results revealed that the SLC7A11/GSH/GPX4 pathway and iron homeostasis were involved in EBBP‐mediated protective effects against DOX‐induced ferroptosis.

The ISR is an adaptive process that helps organisms cope with various stressful environments, such as Endoplasmic reticulum stress, glucose and amino acid deficiency, hypoxia, viral infection, and reactive oxygen species (ROS). Upon activation of the ISR, four upstream kinases, including HRI, PKR, PERK, and GCN2, activate the phosphorylation of eIF2α, leading to a global attenuation of protein translation.^[^ ^21^ ^]^ Simultaneously, several key ISR targets, such as ATF4, are selectively upregulated to enhance protein‐folding capacity and mitigate oxidative stress by detoxifying ROS. Previous studies have demonstrated that the ISR plays a crucial role in maintaining cardiac homeostasis. Unlike the other three kinases, studies have demonstrated that the PERK branch of the ISR has been shown to mitigate myocardial ischemia‐reperfusion injury and pathological cardiac hypertrophy,^[^ ^24^, ^25^ ^]^ establishing its cardioprotective potential. Our investigation reveals a previously uncharacterized role for PERK signaling in doxorubicin‐induced cardiomyopathy. Mechanistically, EBBP enhances PERK activation, and pharmacological inhibition of PERK completely abolishes EBBP‐mediated cardioprotection. Notably, we extend these findings by demonstrating ISR's regulatory intersection with ferroptosis pathways. Our results further demonstrate that pharmacological inhibition of PERK abolishes the cardioprotective effect of EBBP against doxorubicin‐induced ferroptosis in the myocardium. Thus, EBBP protects against ferroptosis in doxorubicin‐induced cardiomyopathy via the PERK‐mediated integrated stress response.

ATF4 is a central orchestrator within the ISR signaling network, coordinating transcriptional activation of adaptive programs governing amino acid metabolism and antioxidant defense mechanisms.^[^ ^20^ ^]^ Emerging evidence reveals complex regulatory interactions between ATF4 and ferroptosis pathways across pathophysiological contexts. While Saini et al. demonstrated PERK/ATF4‐mediated ferroptosis potentiation through SLC7A11 suppression in colorectal cancer models,^[^ ^26^ ^]^ parallel investigations by He et al. identified that the ATF4–SLC7A11 axis regulates glutathione metabolism and suppresses ferroptosis, thereby inhibiting liver damage and cancer progression.^[^ ^53^ ^]^ Nrf2, a master transcription factor, maintains cell homeostasis under stress conditions. Under homeostatic conditions, Nrf2 undergoes ubiquitylation and is directed toward proteasomal degradation. Oxidative challenge triggers Nrf2 stabilization and nuclear translocation, activating cytoprotective gene networks.^[^ ^54^ ^]^ In fact, the effect of Nrf2 on DoIC has been studied. PRMT4 aggravates DOX‐induced cardiac injury and ferroptosis by inhibiting Nrf2/GPX4 pathway.^[^ ^17^ ^]^ PGE2/EP1 suppressed DOX‐induced cardiomyopathy through the PKC/Nrf2/SLC7A11 axis.^[^ ^49^ ^]^ Thus, Nrf2 mitigates DOX‐induced cardiomyopathy by reducing ferroptosis. Our investigation delineates novel regulatory crosstalk between PERK‐mediated ISR and Nrf2 signaling in anthracycline‐induced cardiotoxicity. EBBP overexpression promotes the activation of PERK/ATF4 and phosphorylation of Nrf2 in DOX‐treated cardiomyocytes. In response to ISR, PERK can also phosphorylate Nrf2 directly, promoting its translocation to nuclear to maintain redox homeostasis. Crucially, pharmacological PERK inhibition with GSK2606414 completely abolished EBBP‐induced Nrf2 phosphorylation and subsequent nuclear accumulation. Therefore, ATF4 and Nrf2 are critical downstream effectors of the PERK and EBBP protected against DOX‐induced ferroptosis via the PERK‐eIF2α‐ATF4/Nrf2 axis mediated‐ISR.

As an E3 ubiquitin ligase, EBBP is involves in the posttranslational modification of specific target proteins. Nonetheless, our experiments revealed that EBBP did not bind directly to PERK. GRP78 belongs to heat shock protein 70 family.^[^ ^55^ ^]^ It binds to PERK, thereby inhibiting PERK activity by retaining it within the ER membrane under non‐stressed cellular conditions. Under endoplasmic reticulum (ER) stress, GRP78 dissociates from PERK, leading to the activation of the PERK branch of the ISR. Our investigation identified GRP78 as a novel EBBP substrate and revealed that EBBP catalyzes the K63‐linked ubiquitination of GRP78. K63‐linked polyubiquitin can regulate the interaction, location, and activity of proteins.^[^ ^56^ ^]^ Our study further demonstrated that EBBP‐mediated K63‐linked ubiquitination of GRP78 reduced the repressive interaction between GRP78 and PERK, thereby activating PERK.

The dual role of ferroptosis in both promoting tumor cell death and exacerbating doxorubicin‐induced cardiotoxicity highlights the critical need for tissue‐specific therapeutic targets. Our identification of EBBP as a ferroptosis suppressor in the heart provides a promising theoretical foundation for developing cardiac‐protective therapies without compromising doxorubicin's chemotherapeutic efficacy. Our mechanistic elucidation of EBBP‐mediated K63‐linked polyubiquitination of GRP78 reveals a new therapeutic axis. This posttranslational modification dynamically regulates the GRP78‐PERK interaction, representing a finely tunable target for pharmacological intervention. The EBBP‐GRP78 interface could be targeted by (a) small molecule mimics of EBBP's ubiquitination function, (b) compounds stabilizing the ubiquitinated GRP78 conformation, or (c) biologics modulating this protein interaction. This upstream regulatory mechanism offers greater specificity and reduced risk of pathway overactivation than directly targeting PERK or downstream effectors.

In summary, this study revealed that EBBP alleviates anthracycline‐induced cardiotoxicity by suppressing cardiomyocyte ferroptosis. Mechanistically, EBBP interacts with GRP78 to facilitate its K63‐linked ubiquitination, subsequently activating the PERK/eIF2α axis of the ISR. This signaling cascade culminates in activating downstream effectors ATF4 and Nrf2, which transcriptionally upregulate the SLC7A11/GSH/GPX4 antioxidant axis while concurrently mitigating iron overload. These findings provide a new mechanism of anthracycline‐induced cardiomyopathy, and EBBP may be a potential target for alleviating anthracycline‐induced cardiac injury and heart failure.

## 4 Experimental Section

### Animal Experiments

All animal experiments received approval from the Animal Experimentation Committee at Huazhong University of Science and Technology (IACUC Number: 4391). Wild‐type C57BL/6 mice, aged 8–10 weeks, were procured from Vital River Laboratory Animal Technology Co. Ltd. (Beijing, China) and were maintained at specific pathogen‐free conditions, with a 12‐h light/dark cycle, a room temperature of 22–23 °C, and humidity levels ranging from 40% to 60%.

To achieve cardiac‐specific overexpression or knockdown of EBBP in vivo, an adeno‐associated virus 9 (AAV9) containing EBBP under the cTnT promoter (AAV‐EBBP, with a viral genome concentration of 2 × 10^11 per animal) or shRNA targeting EBBP (AAV‐shEBBP, 2 × 10^11 viral genome per animal) was delivered with a tail vein injection. Control groups received AAV‐NC or AAV‐shNC.

Two weeks after AAV9 injection, a model of doxorubicin‐induced cardiotoxicity (DoIC) was established as previously described.^[^ ^17^ ^]^ Specifically, mice were subjected to a single intraperitoneal injection of doxorubicin (DOX) at a dosage of 15 mg kg^−1^ (Cat#HY‐15142, MedChemExpress) or saline at the indicated times. To inhibit ferroptosis, mice were subjected to daily intraperitoneal injections of Fer‐1 (1 mg kg^−1^; Cat#100579, MedChemExpress) throughout the DOX treatment period. To inhibit PERK activity, mice received a daily oral gavage of PERK inhibitor GSK2606414 (50 mg kg^−1^, Cat#18072, MedChem Express) during the DOX treatment.

Following echocardiographic evaluations of cardiac function, mice were euthanized for systematic tissue collection. Body weights were measured, and blood samples as well as cardiac tissues were harvested for subsequent detailed analysis. This is a list of sequences targeting mouse shEBBP: m‐shEBBP‐1, 5′‐ACCTGCATGGTGAACTACT‐3′, m‐shEBBP‐2, 5′‐CGCAAGTATAGGACCTCGAAA‐3′.

### Echocardiography

Cardiac function was evaluated both prior to and seven days after doxorubicin administration. Specifically, mice were anesthetized with 2% isoflurane, and cardiac function was assessed using a 2D‐guided M‐mode echocardiography imaging system (Vevo 770, VisualSonic, Canada). The left ventricular ejection fraction (LVEF) and fractional shortening (FS) were subsequently calculated using VisualSonic software.

### Histological Analysis

The heart tissue samples were fixed with 4% paraformaldehyde and subsequently embedded in paraffin. Then, heart tissues were deparaffinized and rehydrated through a gradient xylene and ethanol. Tissue sections were then subjected to Hematoxylin & Eosin (H&E) and Masson's trichrome staining to assess cardiac morphology and quantify fibrotic areas. Furthermore, cardiac sections were stained for EBBP (PA5‐104489, Invitrogen) and 4‐HNE (ab46545, Abcam) utilizing an immunohistochemistry kit.

### Immunofluorescence Staining

Heart tissue sections underwent deparaffinization, rehydration, and washing with PBS, followed by antigen retrieval. Neonatal rat cardiomyocytes (NRCMs) were fixed in 4% paraformaldehyde (PFA) for 15 min and permeabilized with 0.1% Triton X‐100. Both heart sections and NRCMs were subjected to blocking with 5% goat serum at room temperature for 1 h. Then, they were incubated overnight at 4 °C with primary antibodies, specifically EBBP (sc‐398851, Santa Cruz) and cTnT (ab8295, Abcam). Following primary incubation, the slides were incubated with fluorescent secondary antibodies for 1 h at room temperature. Nuclei were then stained with DAPI (Servicebio, G1012) for 10 min, with image acquisition performed on a laser scanning confocal microscope (Nikon).

### TUNEL Staining

Intracellular DNA fragments were identified in paraffin‐embedded heart sections and H9c2 cells using the TUNEL BrightRed Apoptosis Detection Kit (Vazyme, China) following the manufacturer's protocol. Briefly, cardiac tissue sections or fixed cells were permeabilized, incubated with TUNEL reaction mixture for 60 min at 37 °C in the dark, and counterstained with DAPI to visualize nuclei. Fluorescent images were acquired using an Olympus fluorescence microscope under standardized exposure conditions. The percentage of TUNEL‐positive cells (red fluorescence) was quantified relative to total nuclei (blue fluorescence) using ImageJ software.

### Transmission Electron Microscopy

Heart tissues were carefully dissected into small pieces (1–2 mm^3^) and fixed in freshly prepared 2.5% glutaraldehyde solution containing 0.1 M sodium cacodylate buffer (pH 7.4) at room temperature for 2 h. Following primary fixation, samples were transferred to 4 °C for overnight stabilization. The fixed tissues were then subjected to standard epoxy resin embedding procedures. Ultrathin sections (90 nm thickness) were obtained using an ultramicrotome, collected on copper grids, and examined using a Hitachi HT7800 transmission electron microscope (Hitachi, Japan).

### Biochemical Detection

Serum samples were collected, and the level of creatine kinase isoenzymes (CK‐MB), lactate dehydrogenase (LDH), and aspartate aminotransferase (AST) were quantified using specific assay kits (Nanjing Jiancheng).

### Cell Culture and Treatment

Neonatal rat cardiomyocytes (NRCMs) were isolated from the hearts of Sprague‐Dawley rats aged 1–3 days, following established protocols.^[^ ^57^ ^]^ In summary, the neonatal hearts (postnatal days 1–3) were excised, minced, and subsequently subjected to digestion using 0.1% type II collagenase (#LS004174, Worthington Biochemical, USA) at 37 °C. The resulting digestion solution containing free cells, was then centrifuged via a discontinuous Percoll gradient to isolate NRCMs. H9c2 rat cardiomyocytes and HEK293T cells were procured from Pricella (Wuhan, China). They were cultured in DMEM enriched with 10 % fetal bovine serum (FBS) and 1 % penicillin‐streptomycin solution, maintained at 37 °C in an incubator with 5% CO_2_.

DOX was administered at varying concentrations (0–5 µm) for a duration of 24 h or at a concentration of 1 µm for different time intervals (0–48 h). Ferrostatin‐1 (Fer‐1, 2 µm, ferroptosis inhibitor, MedChem Express), deferoxamine (DFO, 100 µm, iron chelator, MedChem Express), necrostatin‐1S (Nec‐1, 30 µm, necroptosis inhibitor, MedChem Express), Z‐VAD‐FMK (Z‐VAD, 40 µm, apoptosis inhibitor, MedChem Express), belnacasan (5 µm, caspase1 inhibitor, MedChem Express), 3‐MA (10 µm, autophagy inhibitor, MedChem Express), or Rapamycin (20 nm, autophagy activator, MedChem Express) applied for 2 h prior to DOX treatment. The PERK inhibitor GSK2606414 (2 µm, Cat#18072, MedChem Express) was also administered for 2 h before the DOX exposure. For the determination of protein half‐life, MG132 (20 µm, Cat#13259, MedChem Express) and cycloheximide (CHX) (100 µm, Cat#12320, MedChem Express) were introduced to the cells.

Adenovirus encoding control or EBBP (Ad‐Null or Ad‐EBBP), as well as scramble shRNA or EBBP shRNA (Ad‐shNC or Ad‐shEBBP), were used to achieve the overexpression or knockdown of EBBP, respectively. Cells were subsequently stimulated with DOX for 24 h. The following is a list of the targeting sequences of shRNA that were used against rat EBBP: r‐EBBP‐1,5′‐GGCTTGGCATCTATGTAAA‐3′; r‐EBBP‐2, 5′‐GAGCAGAAGCTCAAGTTGA‐3′.

### Cell Viability Assay

The cell viability was evaluated utilizing the Cell Counting Kit‐8 (Cat#C0042, Beyotime Biotechnology, China). Briefly, 5000 cells per well were seeded into 96‐well plates. Following the various treatments previously outlined, 10 µL CCK‐8 reagent was added to each well and incubated for 2 h at 37 °C. Subsequently, the absorbance was measured at a wavelength of 450 nm.

### ELISA

The concentrations of IL‐1β, IL‐6, and TNF‐α were determined in both H9c2 cell culture supernatants and cardiac tissue homogenates using commercial ELISA kits (IL‐1β/IL‐6/TNF‐α ELISA Kit, Abclonal, China) according to the manufacturer's protocols. For cellular experiments, culture supernatants were collected after treatment and centrifuged at 1000 × g for 10 min to remove debris. Cardiac tissues were homogenized in ice‐cold PBS containing protease inhibitors and centrifuged at 12 000 × g for 15 min at 4 °C to obtain clear supernatants. All samples were analyzed in duplicate and appropriate standard curves included in each assay plate. Absorbance measurements were performed at 450 nm with a reference wavelength of 570 nm using TECAN Infinite F50 (TECAN, Switzerland).

### Lipid Peroxidation Measurement

The cells were cultured in six‐well plate. Following the specified treatment, cells were incubated with 5 µm C11‐BODIPY (581/591) (Cat# D3861, Invitrogen) for 30 min at 37 °C in the dark. Subsequently, the samples were examined by flow cytometry (BD FACS Calibur). The resulting data were analyzed utilizing FlowJo version 10.0 software.

### Measurement of MDA Content

The levels of malondialdehyde (MDA) in serum, cardiac tissue, and cellular samples were measured utilizing the MDA detection kit (BC0025, Solarbio, China).

### Iron Measurement

Cellular levels of ferrous iron (Fe^2+^) were assessed utilizing FerroOrange staining at a concentration of 1 µm (Catalog #F374, DOJINDO, Japan). The quantification of iron levels in heart tissue and cells was conducted employing the Iron Assay Kit (Cat#I219, DOJINDO, Japan) according to the manufacturer's instructions.

### GSH and GSH/GSSG Measurement

The glutathione (GSH) to oxidized glutathione (GSSG) ratio in cardiac tissue and cardiomyocytes was quantified utilizing the GSH and GSSG assay kit (Cat#S0053, Beyotime, China).

### DHE Staining

Superoxide anion levels were assessed by dihydroethidium (DHE) fluorescence staining (S0063, Beyotime Biotechnology, China) following established protocols. Briefly, 5 µm thick frozen heart tissue sections were incubated with 2 µm DHE solution in the dark at room temperature for 30 min. For cellular experiments, H9c2 cells were seeded and cultured in 24‐well plates. Following the indicated treatments, cells were incubated with 2 µm DHE solution at 37 °C for 30 min. Fluorescence imaging was subsequently performed using an Olympus fluorescence microscope.

### Analysis of Mitochondrial Membrane Potential

The mitochondrial membrane potential was measured in H9c2 cells utilizing JC‐1 staining (Cat#2003S, Beyotime, China). In summary, cells were incubated with the JC‐1 working solution for 30 min at 37 °C. Additionally, Hoechst 33342 was employed for nuclear staining. The results were quantitatively presented based on the fluorescence intensity ratio between aggregates (red) and monomers (green).

### SDS‐PAGE and Western Blot Assay

Total proteins were extracted from cardiac tissues or cells utilizing RIPA lysis buffer supplemented with protease and phosphatase inhibitors. Additionally, nuclear proteins were extracted employing a nuclear protein extraction kit (Cat#EX1470, Solarbio, China). A BCA protein assay kit (Thermo Scientific) was used to measure the protein content. Subsequently, the proteins underwent electrophoresis via SDS‐PAGE and were transferred to PVDF membranes (Millipore, IPVH00010). After blocking with 5% non‐fat milk for 1 h at room temperature, the membranes were incubated overnight at 4 °C with the appropriate primary antibodies. After washing, the membranes were incubated with HRP‐conjugated secondary antibodies, and the resulting bands were visualized using a Clinx imaging system (Shanghai). The primary antibodies are listed in Table S1 (Supporting Information).

### RNA Extraction and Real‐Time PCR

Total RNA was extracted from hearts or cells utilizing TRIzol reagent (Vazyme). The extracted RNA was subjected to reverse transcription reactions using the PrimeScript RT Master Mix Kit (TaKaRa, RR036A). Following this, real‐time quantitative PCR was conducted employing ChamQTM Universal SYBR® qPCR Master Mix (Vazyme) on an ABI 7500 Real‐Time PCR system (Thermo Fisher Scientific). All of the primer sequences that were used in the research are included in Table S2 (Supporting Information).

### Bulk RNA Sequencing

Total RNA was isolated from murine cardiac tissues in AAV‐NC and AAV‐EBBP groups following DOX treatment using established protocols. Subsequent RNA integrity assessment and high‐throughput sequencing were executed by SpecAlly (Wuhan, China). Bioinformatics analysis involved DESeq2‐based normalization and differential expression profiling to identify transcripts that met stringent significance criteria (|log2FC| ≥ 1, adjusted *p* value < 0.05) across comparative groups. KEGG pathway enrichment analysis was conducted on differentially expressed genes (DEGs) using the R package clusterProfler to identify statistically significant functional annotations.

### Plasmid Construction

Plasmids containing both full‐length and truncated forms of EBBP, GRP78, and PERK were constructed by cloning from complementary DNA (cDNA) and subsequently inserted into the pcDNA3.1 vector. Additionally, Myc‐tagged ubiquitin variant plasmids (Myc‐Ub, Myc‐Ub‐K6O, Myc‐Ub‐K11O, Myc‐Ub‐K27O, Myc‐Ub‐K48O, Myc‐Ub‐K63O, Myc‐Ub‐K48R, Myc‐Ub‐K63O Myc‐Ub‐K63R) were procured from MiaolingBio (Wuhan, China). The transfection of these plasmids into HEK293T cells was performed using PEI transfection reagent once the cells reached ≈80% confluence.

### Luciferase Reporter Assay

The rat EBBP promoter was PCR‐amplified and cloned into the pGL3‐Basic vector (Promega). HEK293T cells were seeded into 24‐well plates and co‐transfected at 70–80% confluence with 500 ng pGL3‐EBBP plasmid along with transcription factor expression vectors, plus 50 ng of the internal control plasmid pRL‐TK (Renilla luciferase reporter plasmid, Promega) using PEI transfection reagent. Following 48 h of incubation, cells were harvested and lysed for dual‐luciferase activity measurement using the TransDetect® Double‐Luciferase Reporter Assay Kit (TransGene Biotech, China). Firefly and Renilla luciferase activities were quantified sequentially using the Dual‐Luciferase Reporter Assay System (Promega, USA).

### Molecular Docking Analysis

The 3D structures of GRP78 (UniProt ID P11021) and EBBP (UniProt ID O95361) were retrieved from the UniProt database. Protein‐protein docking was performed using the HDOCK server to predict the interaction mode between GRP78 and EBBP. The docking results revealed a strong binding affinity between the two proteins, with a calculated binding energy of −224.65 kcal mol^−1^. Analysis of the interaction interface identified potential hydrogen bond formations between adjacent residues, which may contribute to the stabilization of the protein complex. Molecular visualization and interaction pattern analysis were conducted using PyMol software (version 2.3.0, Schrödinger LLC). In the figure, GRP78 protein was shown as a blue color cartoon, and EBBP as red color cartoon with their predicted binding cites shown as colored sticks.

### Co‐Immunoprecipitation

Extraction of cell proteins was carried out as described above. The resulting protein supernatants were incubated with the designated antibodies overnight at 4 °C. This was followed by a 2‐h incubation with protein A/G magnetic beads (MCE, HY‐K0202). The beads were subsequently washed four times with the IP lysis buffer and then boiled in SDS loading buffer for 10 min. Finally, western blotting was conducted as previously described.

### Ubiquitination Assays

Following the transfection of HEK293T cells with the specified plasmids, the cells were treated with MG132 for 6 h before harvest. Then, the cells were collected and lysed using 100 µL of lysis solution. Subsequently, the cells were denatured by heating at 95 °C for a duration of 10 min. Following that, 900 µL of immunoprecipitation (IP) lysis buffer was added to the lysates, which were then subjected to sonication and centrifugation. The resulting samples were incubated with the designated antibodies and protein A/G magnetic beads at 4 °C for a duration of 3 h. Following this incubation, the beads were washed four times with IP buffer and then boiled in SDS loading buffer for 10 min. Finally, western blotting was carried out in accordance with the procedure that had been devised before.

### Statistical Analysis

Statistical analyses were conducted utilizing GraphPad Prism software version 9.0. The data was expressed as mean ± SD. The number of biological replicates (n) for each statistical analysis is specified in the figure legends. The two‐tailed unpaired Student's *t*‐test was utilized for the purpose of comparing two groups, whilst the one‐way analysis of variance (ANOVA) followed by Tukey's multiple comparison test was utilized for the purpose of conducting studies that involved several groups. A *p‐*value< 0.05 was deemed statistically significant.

## Conflict of Interest

The authors declare no conflict of interest.

## Author Contributions

Z.C. and C.C. contributed equally to this work. K.H., C.W., and Z.W. conceived the study. Z.C., C.C., Y.W., and Y.X. performed the animal and cell experiments. R.L., J.S., and L.C. analyzed the data. K.H., C.W., and Z.W. drafted manuscript, and K.H. and C.W. revised the manuscript.

## Supporting information



Supporting Information

## Data Availability

The data that support the findings of this study are available from the corresponding author upon reasonable request.
