# Supplementary material for: EBBP‐Mediated Integrated Stress Response Attenuates Anthracycline‐Induced Cardiotoxicity by Inhibiting the Ferroptosis of Cardiomyocytes
Source: Adv Sci (Weinh). 2025 Jun 10;12(32):e02726. doi: 10.1002/advs.202502726 (PMC12407386; doi:10.1002/advs.202502726)
Supplement: Supplementary file 1 — Supporting Information [file ADVS-12-e02726-s001.docx]

**EBBP‐Mediated Integrated Stress Response Attenuates Anthracycline‐Induced Cardiotoxicity by Inhibiting the Ferroptosis of Cardiomyocytes**

*Zilong Chen ^1,2 #^, Can Chen^1,2 #^, Yichen Wu ^1^, Yinxue Xia**^1,2^, Ruijie Luo^1,2^, Jiangcheng Shu ^4^, Long Chen ^2^, Zhaohui Wang^1,2 *^, Cheng Wang^2,3 *^, Kai Huang^1,2,5,6 *^*

1 Department of Cardiology, Union Hospital, Tongji Medical College, Huazhong University of Science and Technology, Wuhan, 430022, China.

2 Clinic Center of Human Genomic Research, Union Hospital, Tongji Medical College, Huazhong University of Science and Technology, Wuhan, 430022, China.

3 Department of Rheumatology, Union Hospital, Tongji Medical College, Huazhong University of Science and Technology, Wuhan, 430022, China.

4 Department of Geriatrics, Southwest Hospital, Third Military Medical University (Army Medical University), Chongqing, 400038, China.

5 Hubei Key Laboratory of Metabolic Abnormalities and Vascular Aging, Huazhong University of Science and Technology, Wuhan, China.

6 Hubei clinical research center of metabolic and cardiovascular disease, Huazhong University of Science and Technology, Wuhan, China

# Zilong Chen and Can Chen have contributed equally to this work.

Correspondence to:

Zhaohui Wang, Department of Cardiology, Union Hospital, Tongji Medical College, Huazhong University of Science and Technology, 1277 Jiefang Ave, Wuhan, Hubei 430022, China; Email: [1992xh0628@hust.edu.cn](mailto:1992xh0628@hust.edu.cn)

Cheng Wang, Department of Rheumatology, Union Hospital, Tongji Medical College, Huazhong University of Science and Technology, Wuhan, 430022, China; Email: cwangunion@hust.edu.cn

Kai Huang, Department of Cardiology, Union Hospital, Tongji Medical College, Huazhong University of Science and Technology, 1277 Jiefang Ave, Wuhan, Hubei 430022, China; Email: huangkai1@hust.edu.cn


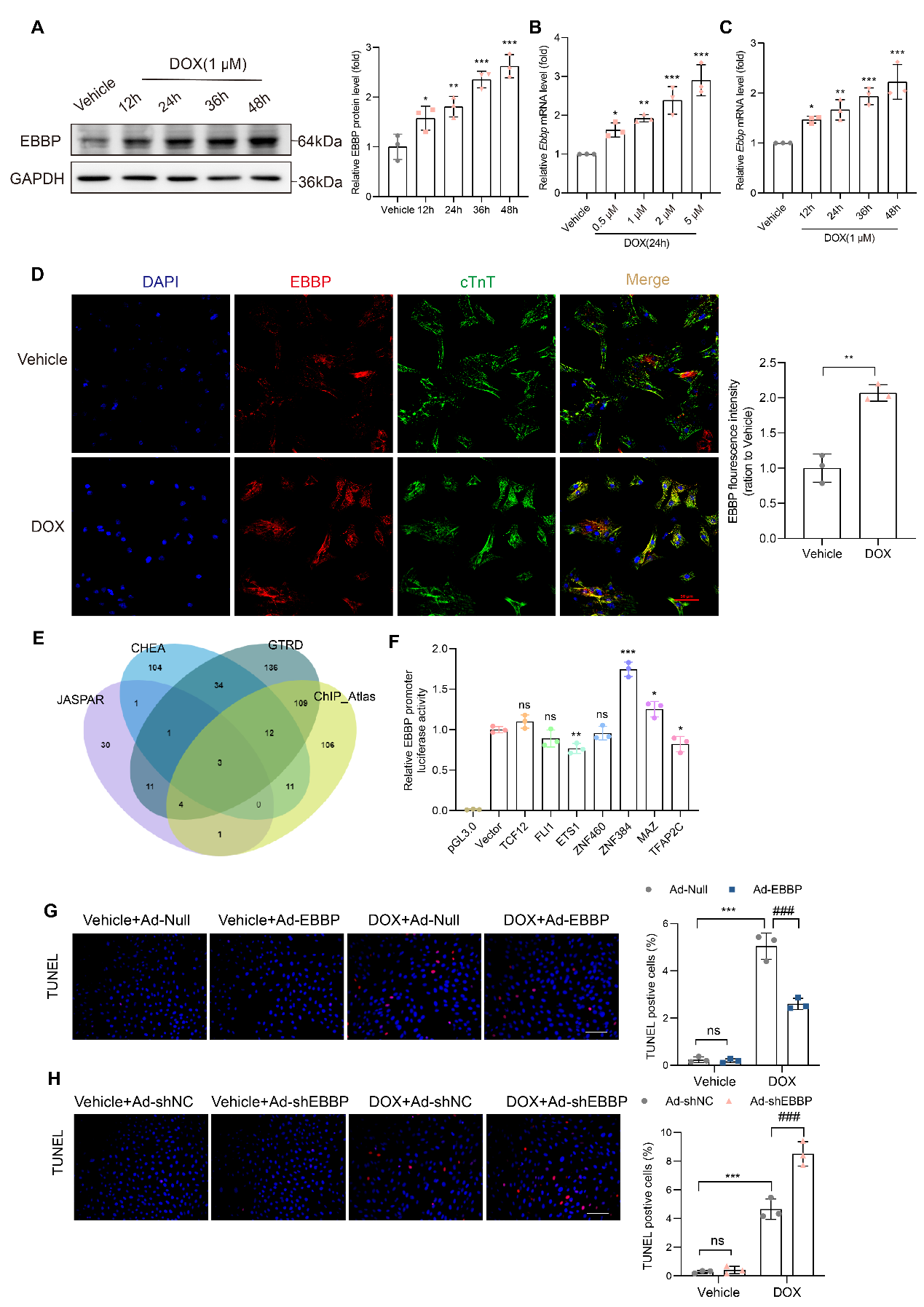


**Figure S1. EBBP is involved in anthracycline-induced cardiotoxicity.**

**A.** Immunoblots and statistical analysis of EBBP in NRCMs treated with DOX (1 μM) at different times (n=3). **B-C.** The mRNA expression levels of EBBP in NRCMs exposed to varying concentrations of doxorubicin for 24 h or incubated with 1 μM DOX for differing durations (n=3). **D.** Representative images and quantitative analysis of EBBP level in NRCMs after DOX treatment using immunofluorescent staining. Red, EBBP; Green, cTnT; Nuclei were stained with DAPI (blue). (scale bars, 50 μm; n = 3). **E**. Venn diagram showing the intersection of putative transcriptional regulators of EBBP predicted by JASPAR, CHEA, GTRD and ChIP-Atlas databases. **F.** Luciferase assay analyzing the effect of putative transcriptional factors on the EBBP promoter (n=3). **G-H.** Following infection with Ad-EBBP or Ad-shEBBP, H9c2 was subjected to a DOX (1 μM) treatment for a duration of 24 hours. Representative images and quantitative analysis of TUNEL positive cells in H9c2 cells (n=3). Values are presented as the mean±SD. **p* < 0.05, ***p* < 0.01 and ****p* < 0.01 vs. Vehicle group; ^###^*p* < 0.001 vs. DOX+ Ad-Null or DOX+ Ad-shNC.

**
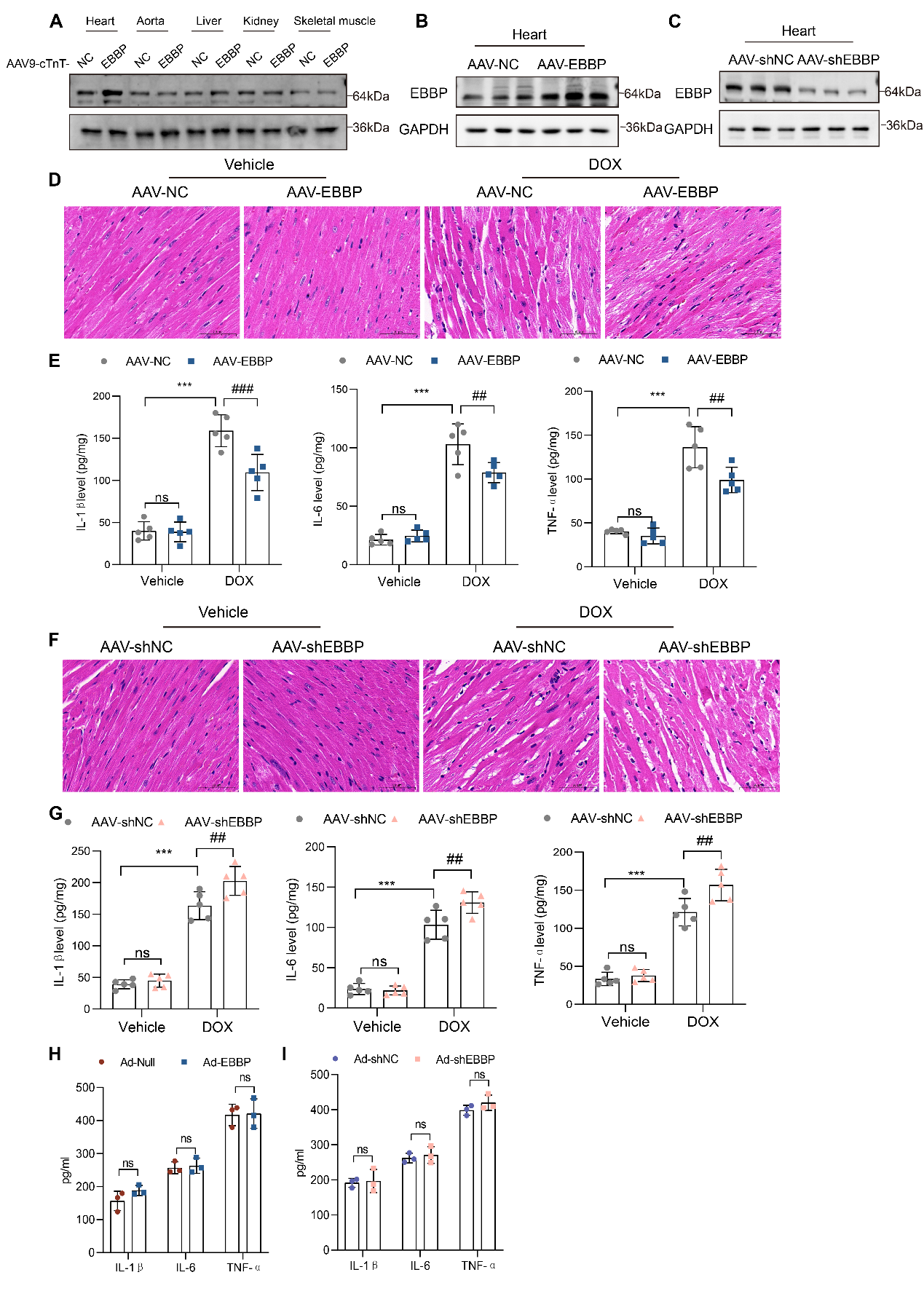
**

**Figure S2. EBBP mitigates doxorubicin-induced myocardial inflammation in vivo, while it exhibits no effect in vitro.**

**A.** Representative immunoblots of EBBP protein in the heart, aorta, liver, kidney, and skeletal muscle from AAV-NC and AAV-EBBP mice. **B.** Representative immunoblots of EBBP protein in the heart from AAV-NC and AAV-EBBP mice. **C.** Representative immunoblots of EBBP protein in the heart from AAV-shNC and AAV-shEBBP mice. **D.** Representative micrographs of H&E staining in the heart administered with AAV-NC or AAV-EBBP (scale bars, 50μm). **E.** The levels of IL-1β, IL-6, and TNF-α in the cardiac tissues administered with AAV-NC or AAV-EBBP (n=5). **F.** Representative micrographs of H&E staining in the heart administered with AAV-shNC or AAV-shEBBP (scale bars, 50μm). **G.** The levels of IL-1β, IL-6, and TNF-α in the cardiac tissues administered with AAV-shNC or AAV-shEBBP (n=5). **H, I.** The levels of IL-1β, IL-6, and TNF-α in the supernatant of DOX-treated H9c2 cells infected with Ad-EBBP or Ad-shEBB were measured (n=3). Values are presented as the mean±SD. ****p* < 0.001 vs. Vehicle + AAV-NC or Vehicle + AAV-shNC; ^##^*p* < 0.01 and ^###^*p* < 0.001 vs. DOX + AAV-NC or DOX + AAV- shNC group.


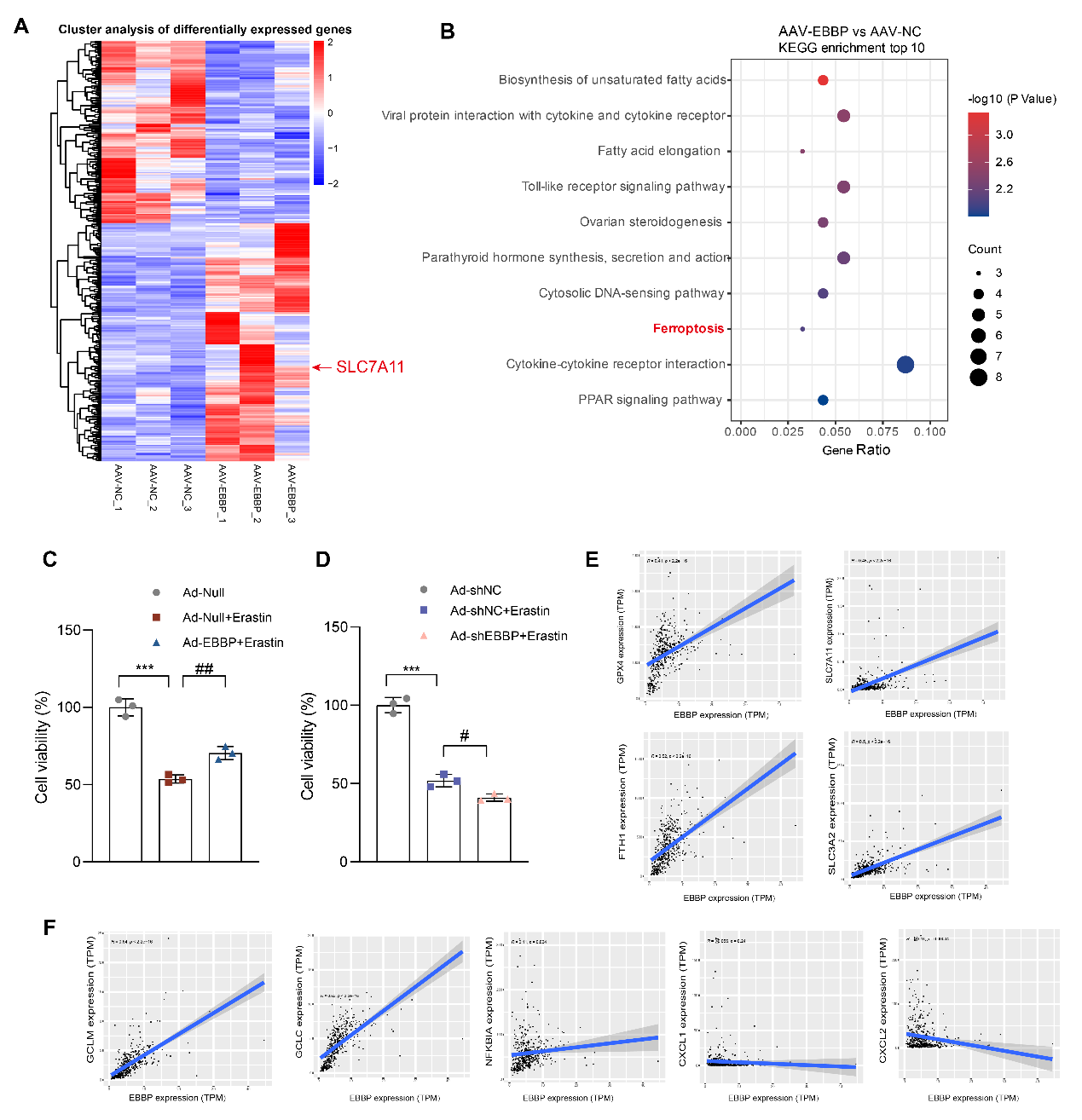


**Figure S3.** **EBBP is implicated in the process of ferroptosis.**

**A.** Heatmap illustrating differentially expressed genes identified through RNA-seq analysis of murine heart tissue from DOX+AAV-NC and DOX+AAV-EBBP group. **B.** KEGG pathway enrichment analysis of DOX-treated murine hearts administered with AAV-NC or AAV-EBBP. **C-D.** H9c2 were infected with Ad-EBBP or Ad-shEBBP and treated with Erastin for 24 h. Then, cell viability was measured by CCK8 (n=3). **E-F.** Correlation analysis between EBBP and key genes involved in KEGG pathways was performed on RNA sequence data from normal human heart specimens in the GTEx database. Values are presented as the mean±SD. **p* < 0.05 and ****p* < 0.001 vs. Vehicle + Ad-Null or Vehicle+ Ad-shNC; ^#^*p* < 0.05, ^##^*p* < 0.01 vs. DOX+ Ad-Null or DOX+ Ad-shNC.


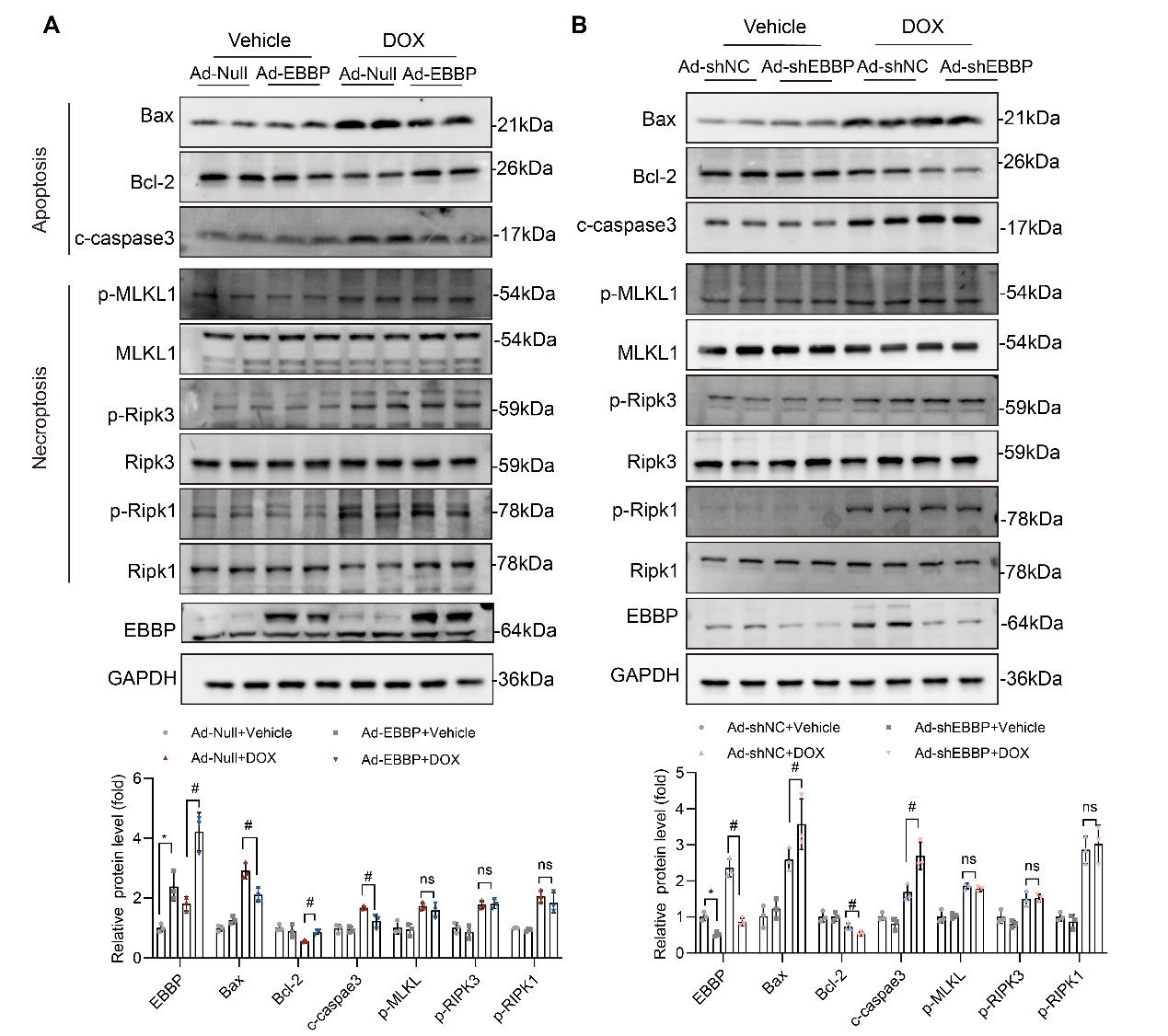


**Figure S4.** **EBBP does not regulate necroptosis but exerts inhibitory effects on apoptosis in DOX-treated H9c2 cells.**

**A-B.** Representative immunoblots and quantitative analysis of the effects of EBBP on apoptosis- and necroptosis-related proteins in DOX-treated H9c2 cells (n=3). Values are presented as the mean±SD. **p* < 0.05 vs. Vehicle + Ad-Null or Vehicle+ Ad-shNC; ^#^*p* < 0.05 vs. DOX+ Ad-Null or DOX+ Ad-shNC.


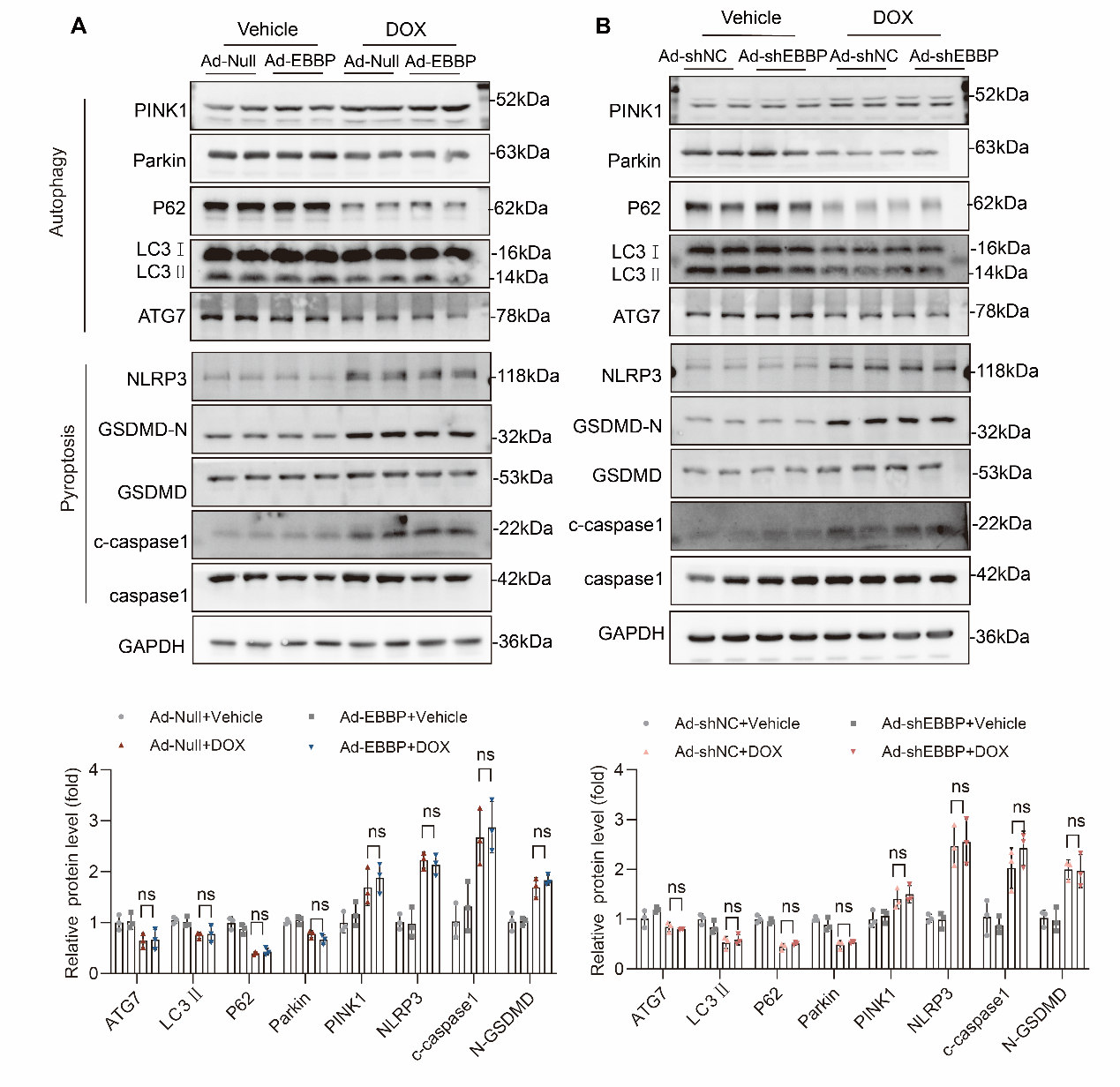


**Figure S5.** **EBBP exhibits no influence on autophagy and pyroptosis in DOX-treated H9c2 cells.**

**A-B.** Representative immunoblots and quantitative analysis of the effects of EBBP on autophagy- and pyroptosis-related proteins in DOX-treated H9c2 cells (n=3). Values are presented as the mean±SD. ns, non-significant.


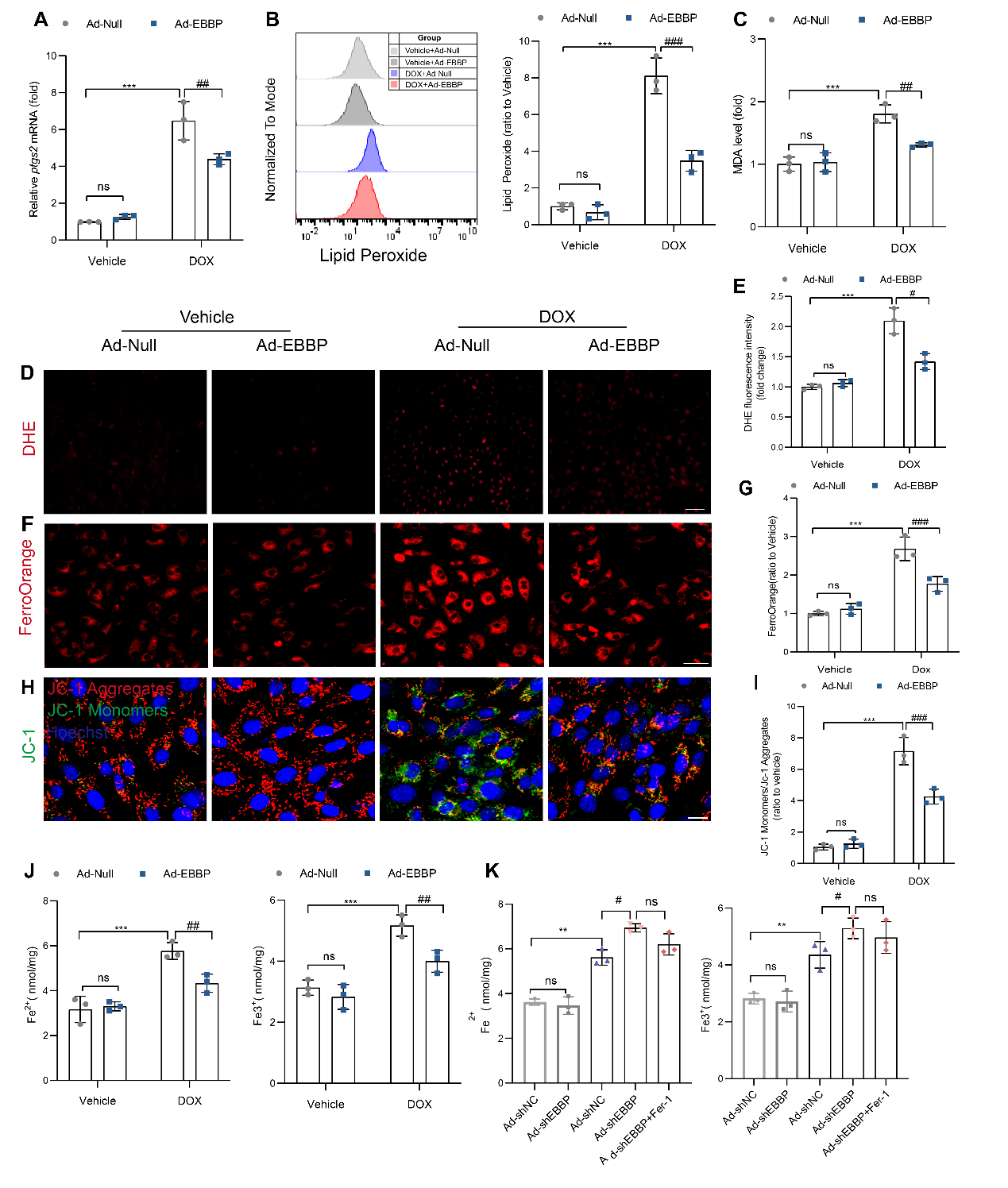


**FigureS6. EBBP ameliorates anthracycline-induced cardiomyocyte ferroptosis in vitro.**

Following infection with Ad-NC or Ad-EBBP, Ad-shNC or Ad-shEBBP, H9c2 was treated with DOX (1 μM) for 24 h. **A.** The mRNA level of Ptgs2 was detected by qRT-PCR (n=3). **B.** Representative pictures and statistical analysis of intracellular lipid peroxide by flow cytometry (n=3). **C.** MDA level in cells (n=3). **D-E.** Representative images and quantification of fluorescent immunohistochemistry staining for DHE in H9c2 cells (scale bars, 100μm, n=3). **F-G.** Representative images and quantification of intracellular Fe^2+^ levels by FerroOrange staining in H9c2 cells (scale bars, 50μm, n=3). **H-I.** Representative images and quantification of mitochondrial membrane potential (JC-1 staining) in H9c2 cells (scale bars, 20μm; n=3). **J-K.** Quantitative analysis of intracellular Fe^2+^ and Fe^3+^ using colorimetric methods in H9c2 cells (n=3). Values are presented as the mean±SD. ***p* < 0.01 and ****p* < 0.001 vs. Vehicle + Ad-Null or Vehicle+ Ad-shNC; ^#^*p* < 0.05, ^##^*p* < 0.01 and ^###^*p* < 0.001 vs. DOX+ Ad-Null or DOX+ Ad-shNC.


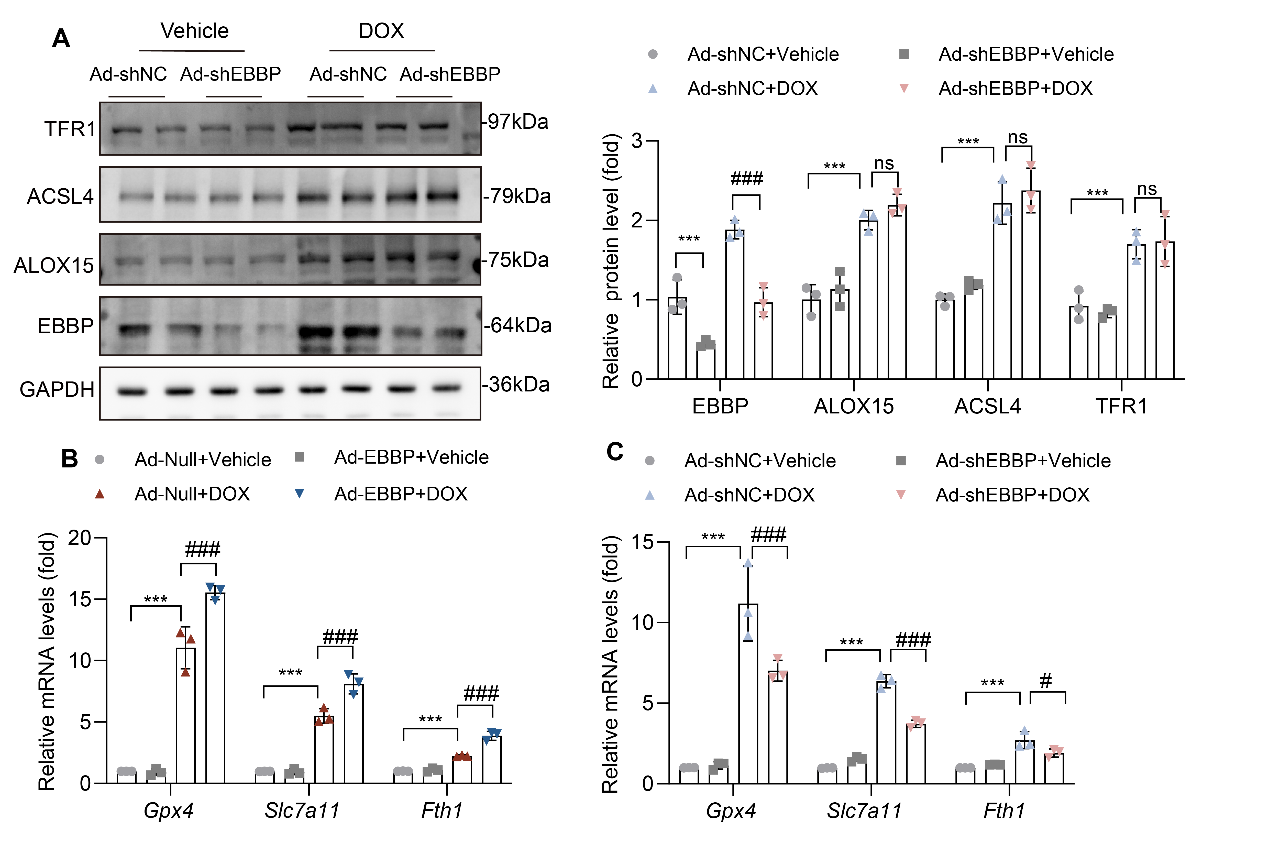


**FigureS7. EBBP upregulates SLC7A11/GPX4 and FTH1 mRNA expression.**

**A.** Representative immunoblots and quantitative analysis of the effects of EBBP on pro-ferroptosis proteins in DOX-treated H9c2 cells (n=3). **B-C.** The mRNA levels of *Slc7a11*, *Gpx4*, and *Fth1* were detected by qRT-PCR (n=3). Values are presented as the mean±SD. ****p* < 0.001 vs. Vehicle + Ad-Null or Vehicle+ Ad-shNC; ^#^*p* < 0.05 and ^###^*p* < 0.001 vs. DOX+ Ad-Null or DOX+ Ad-shNC.

**
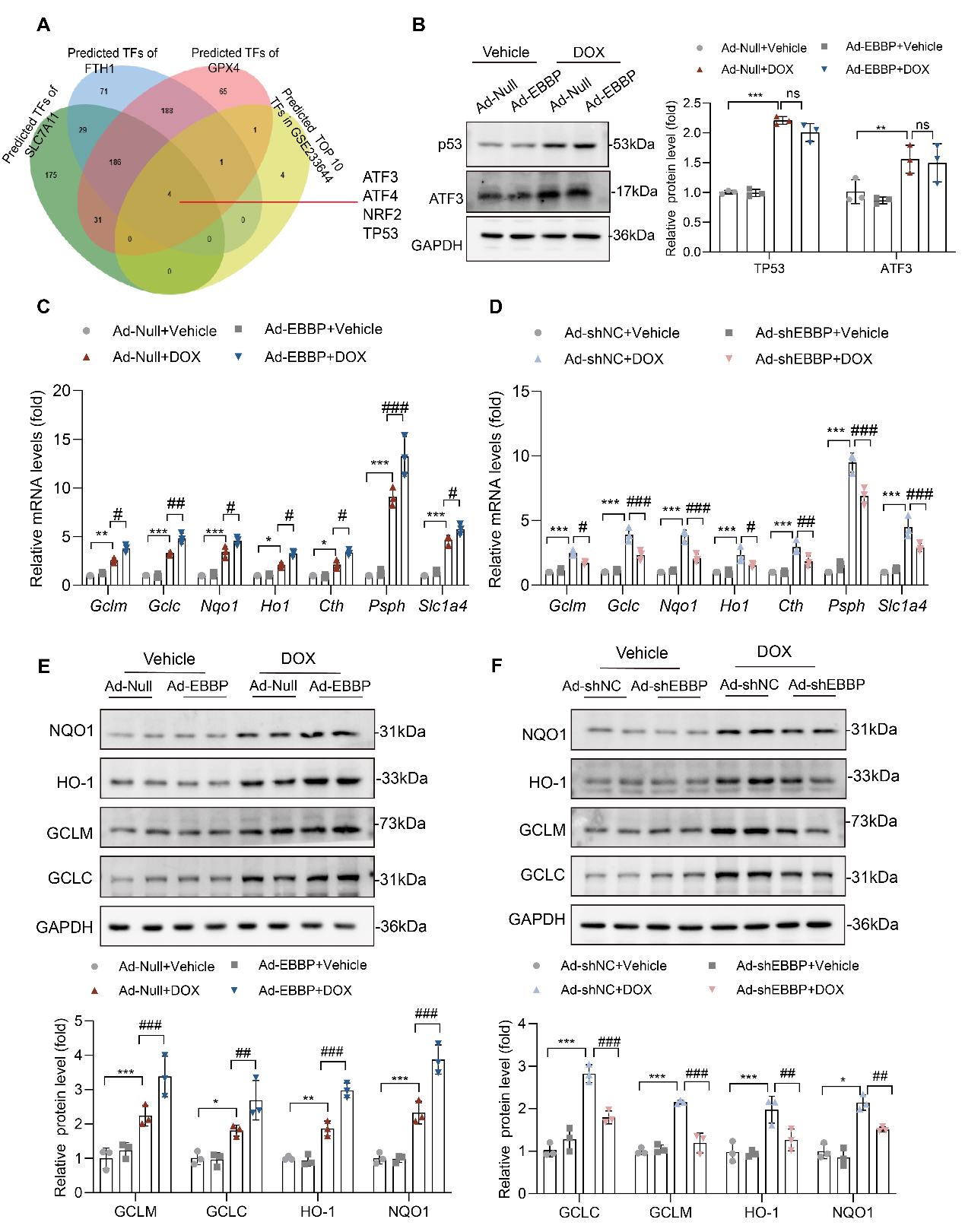
**

**FigureS8. EBBP enhances the transcriptional activation of genes targeted by ATF4 and Nrf2.**

**A.** Venn diagram showing the intersection of putative transcriptional regulators of SLC7A11, GPX4, and FTH1 predicted by the GTRD database and the top 10 regulators implicated in the pathogenesis of doxorubicin-induced cardiomyopathy predicted by the ChEA3 database using GSE233644. **B.** Representative western blots and statistical analysis of P53 and ATF3 in H9c2 cells (n=3). **C-D.** The mRNA levels of major target genes of ATF4 and Nrf2 were detected by qRT-PCR (n=3). **E-F.** Representative immunoblots and quantitative analysis of the target proteins of Nrf2 in DOX-treated H9c2 cells (n=3). Values are presented as the mean±SD. **p* < 0.05, ***p* < 0.01 and ****p* < 0.001 vs. Vehicle + Ad-Null or Vehicle+ Ad-shNC; ^#^*p* < 0.05, ^##^*p* < 0.01 and ^###^*p* < 0.001 vs. DOX+ Ad-Null or DOX+ Ad-shNC.


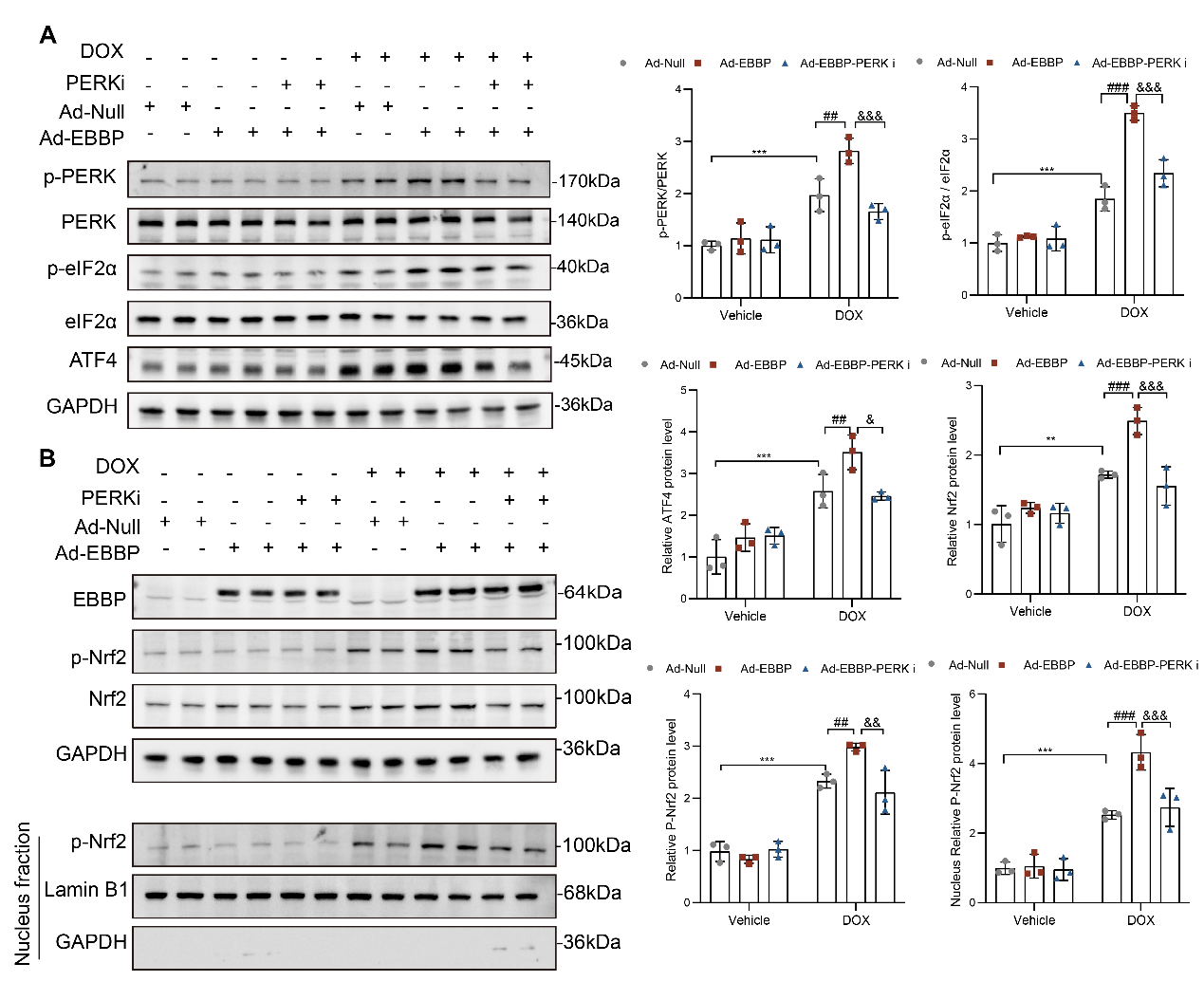


**FigureS9. EBBP promotes the activation of Nrf2 and ATF4 through phosphorylation of PERK.**

**A-B.** Immunoblots and statistical analysis of p-PERK, PERK, p-eIF2α, eIF2α, ATF4, p-Nrf2, and Nrf2 in H9c2 cells infected with Ad-Null or Ad-EBBP and pretreated with the PERK inhibitor GSK2606414 (2 μM) for 2 h followed by stimulation with DOX (n=3). Values are presented as the mean±SD. ***p* < 0.01 and ****p* < 0.001 vs. Vehicle + Ad-Null; ^##^*p* < 0.01 and ^###^*p* < 0.001 vs. DOX+ Ad-Null; ^&^*p* < 0.05, ^&&^*p*< 0.01 and ^&&&^*p* < 0.001 vs. DOX+Ad-EBBP group.


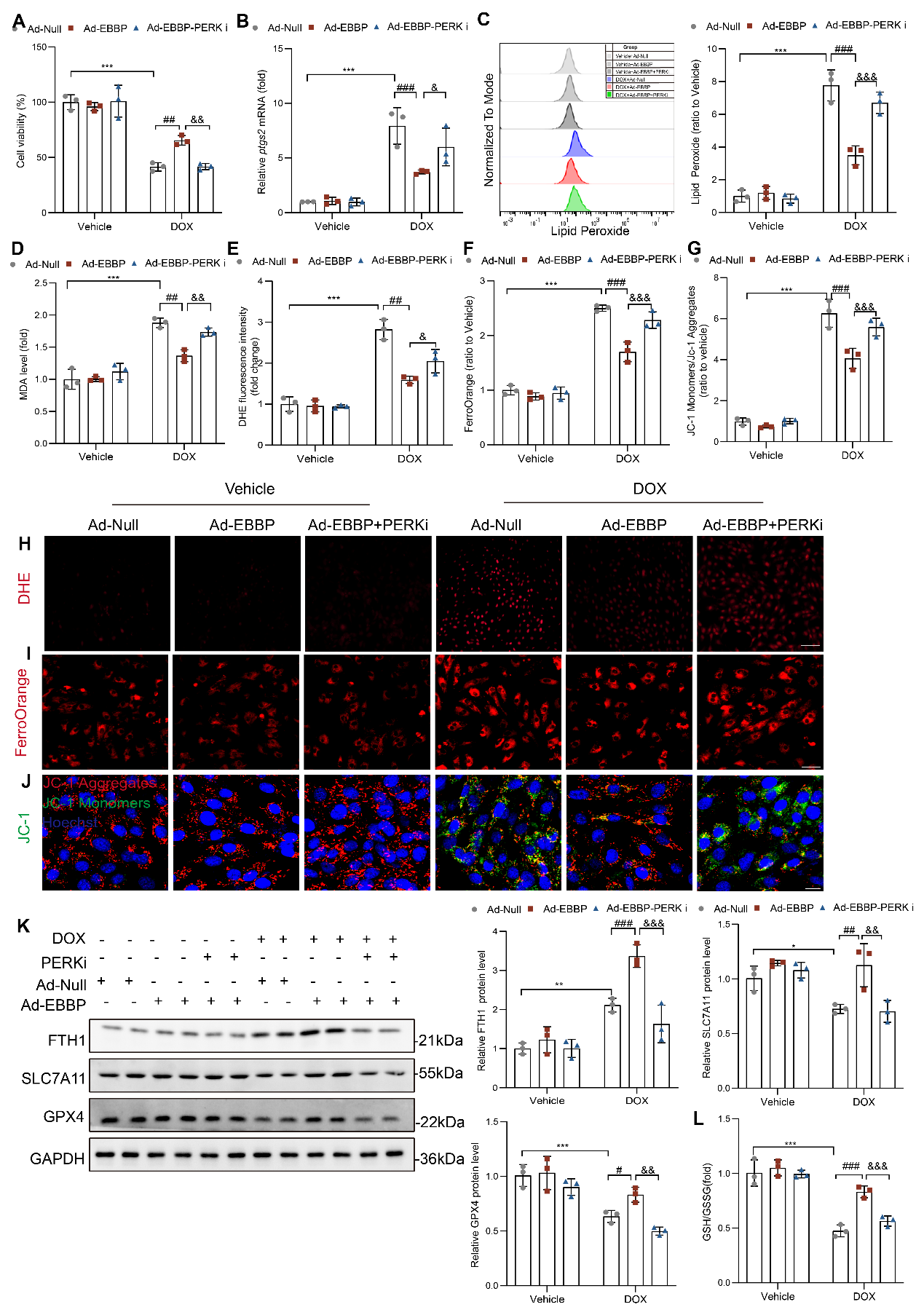


**FigureS10. EBBP attenuates anthracycline-induced cardiomyocyte ferroptosis by PERK-mediated ISR.**

H9c2 cells were infected with Ad-Null or Ad-EBBP and pre-treated with the PERK inhibitor GSK2606414 (2 μM) for 2 h, followed by stimulation with DOX.

**A.** Cell viability was measured by CCK8 (n=3). **B.** qRT-PCR was used to determine the amount of Ptgs2 mRNA (n=3). **C.** Representative pictures and statistical analysis of intracellular lipid peroxide by flow cytometry (n=3). **D.** MDA level in cells (n=3). **E.** Quantification of fluorescent immunohistochemistry staining for DHE in H9c2 cells (n=3). **F.** Quantification of intracellular Fe^2+^ levels (FerroOrange staining) in H9c2 cells (n=3). **G.** Quantification of mitochondrial membrane potential (JC-1 staining) in H9c2 cells (n=3). **H-J.** Representative images of DHE staining (scale bars, 100μm), FerroOrange staining (scale bars, 50μm), and JC-1 staining (scale bars, 20μm). **K.** Immunoblots and statistical analysis of FTH1, SLC7A11, and GPX4 in H9c2 cells (n=3). **L.** GSH/GSSG in H9c2 cells (n=3). Values are presented as the mean±SD. **p* < 0.05, ***p* < 0.01 and ****p* < 0.001 vs. Vehicle + Ad-Null; ^#^*p* < 0.05, ^##^*p* < 0.01 and ^###^*p* < 0.001 vs. DOX+ Ad-Null; ^&&^*p* < 0.01, ^&&&^*p* < 0.001 vs. DOX+Ad-EBBP group.


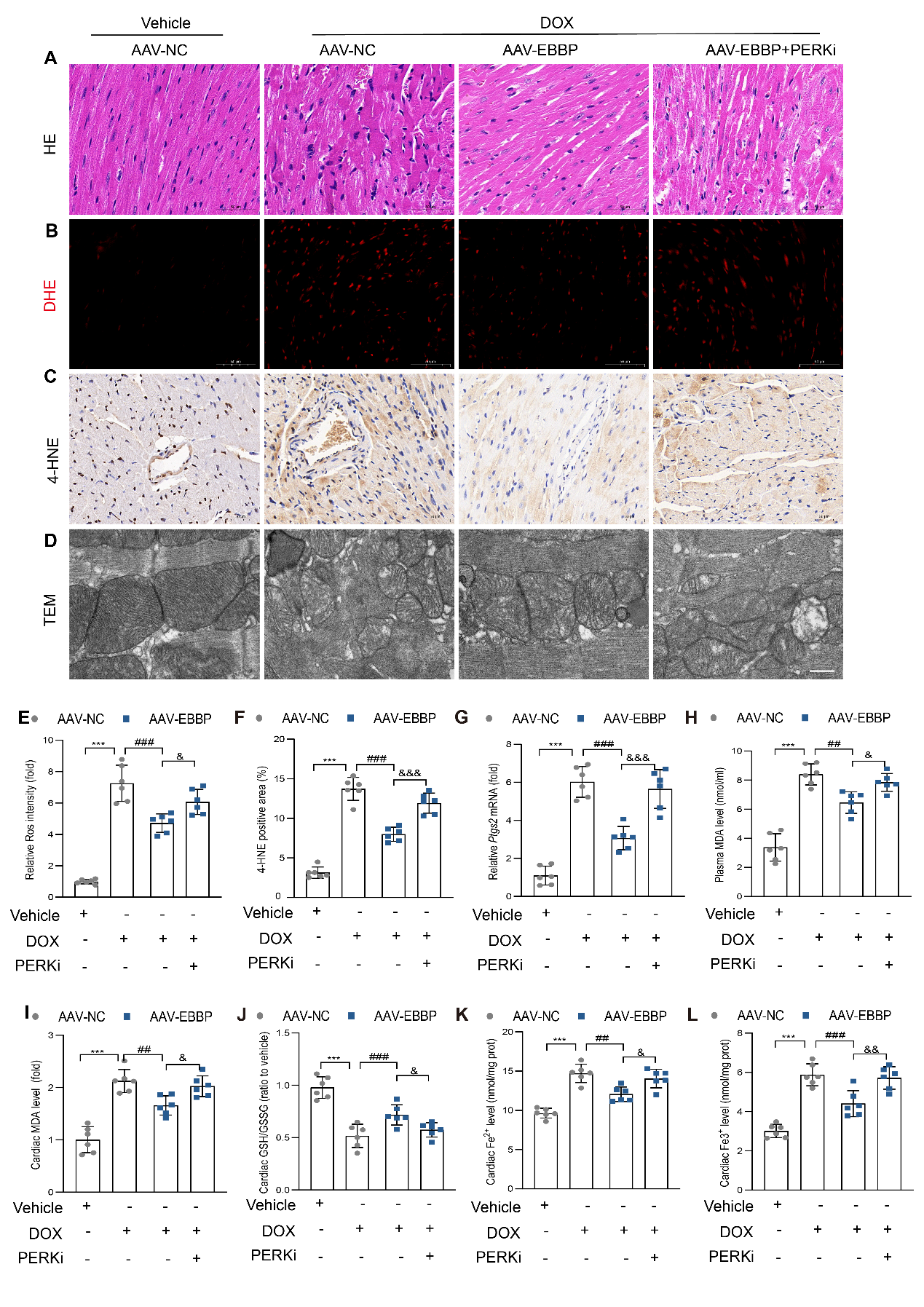


**FigureS11.** **Pharmacological inhibition of PERK attenuates EBBP's cardioprotective effects in anthracycline-induced myocardial ferroptosis.**

**A.** Representative images of H&E staining in cardiac tissues (scale bars, 50μm)**. B.** Representative images of fluorescent immunohistochemistry staining for DHE in mouse myocardium (scale bars, 100μm). **C.** Representative images of immunohistochemistry for 4-HNE in mouse myocardium (scale bars, 50μm). **D.** Representative transmission electron micrographs of cardiac tissues (Scale bar,500 nm). **E.** Quantification of fluorescent immunohistochemistry staining for DHE in mouse myocardium (n=6) **F.** Quantification of immunohistochemistry for 4-HNE in mouse myocardium (n=6). **G.** The mRNA levels of Ptgs2 mRNA in cardiac tissues (n=6). **H.** Plasma MDA level (n = 6). **I.** MDA levels in cardiac tissues (n=6). **J.** GSH/GSSG in cardiac tissues (n=6). **K-L.** Fe^2+^ and Fe^3+^ levels in cardiac tissues (n=6). Values are presented as the mean±SD. ****p* < 0.001 vs. Vehicle + AAV-NC; ^##^*p* < 0.01 and ^###^*p* < 0.001 vs. DOX + AAV-NC group; ^&^*p* < 0.05, ^&&^*p* < 0.01 and ^&&&^*p* < 0.001 vs. DOX + AAV-EBBP group.


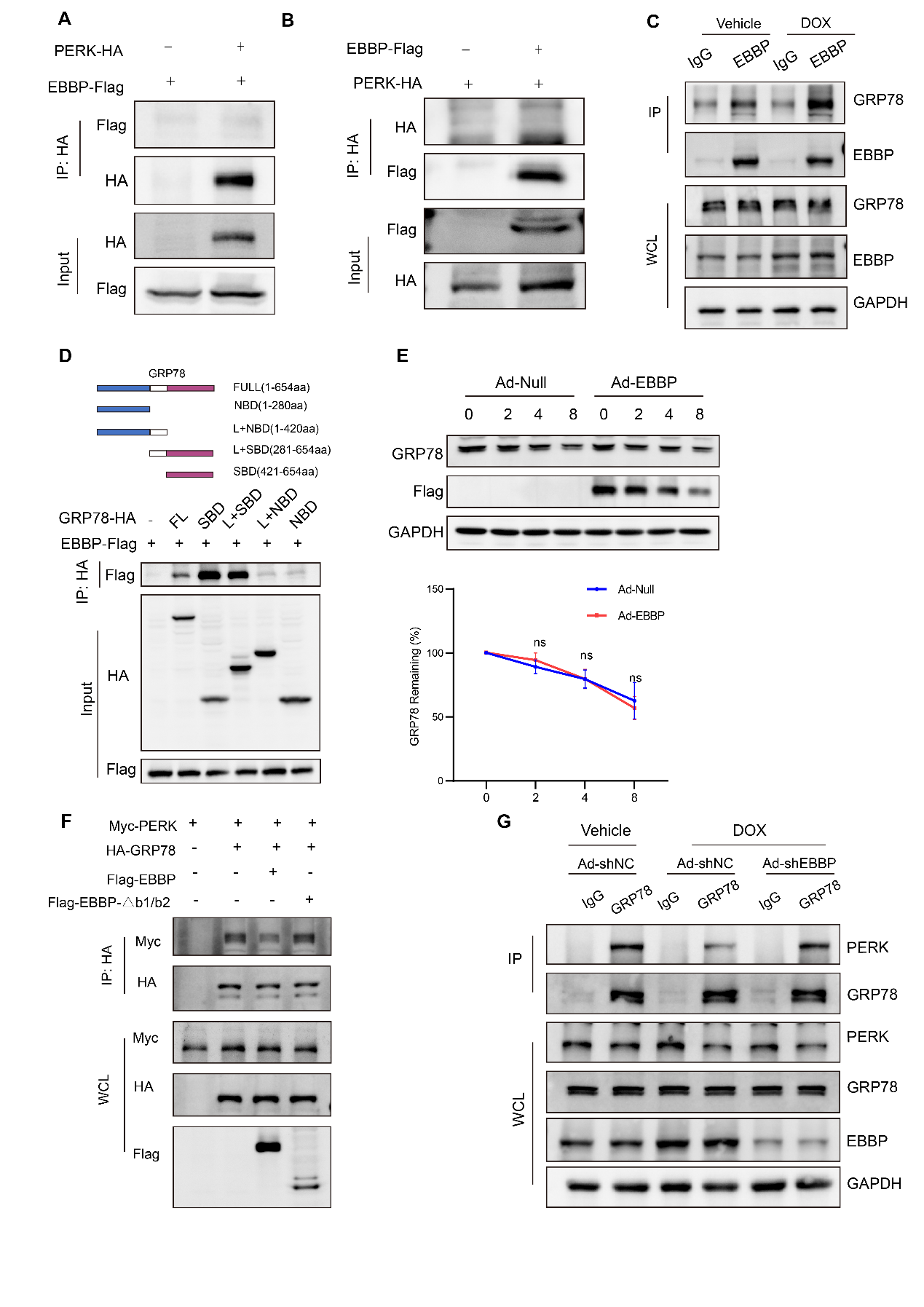


**FigureS12.** **EBBP disrupts the inhibitory interaction between GRP78 and PERK.**

**A-B.** Coimmunoprecipitation (Co-IP) experiments of the interaction between EBBP and PERK in 293T cells. **C.** An endogenous Co-IP study was performed to investigate the interaction between EBBP and GRP78 in NRCMs treated with vehicle or DOX for 24 h. WCL, whole-cell lysis. **D.** A diagrammatic representation of the domains of GRP78 and the shortened mutants is shown. Co-IP analysis of the interaction domains of GRP78 and EBBP. **E.** Immunoblots of GRP78 degradation in H9c2 cells that were infected with Ad-Null or Ad-EBBP and exposed to cycloheximide CHX for indicated times (n=3). **F.** Co-IP assays were performed to analyze the interaction between GRP78 and PERK in 293T cells transfected with Flag-EBBP or Flag-EBBP-△b1/b2. **G.** The endogenous interaction between GRP78 and PERK was investigated by IP analysis in NRCMs infected with Ad-shNC or Ad-shEBBP and treated with vehicle or DOX for 24 h.


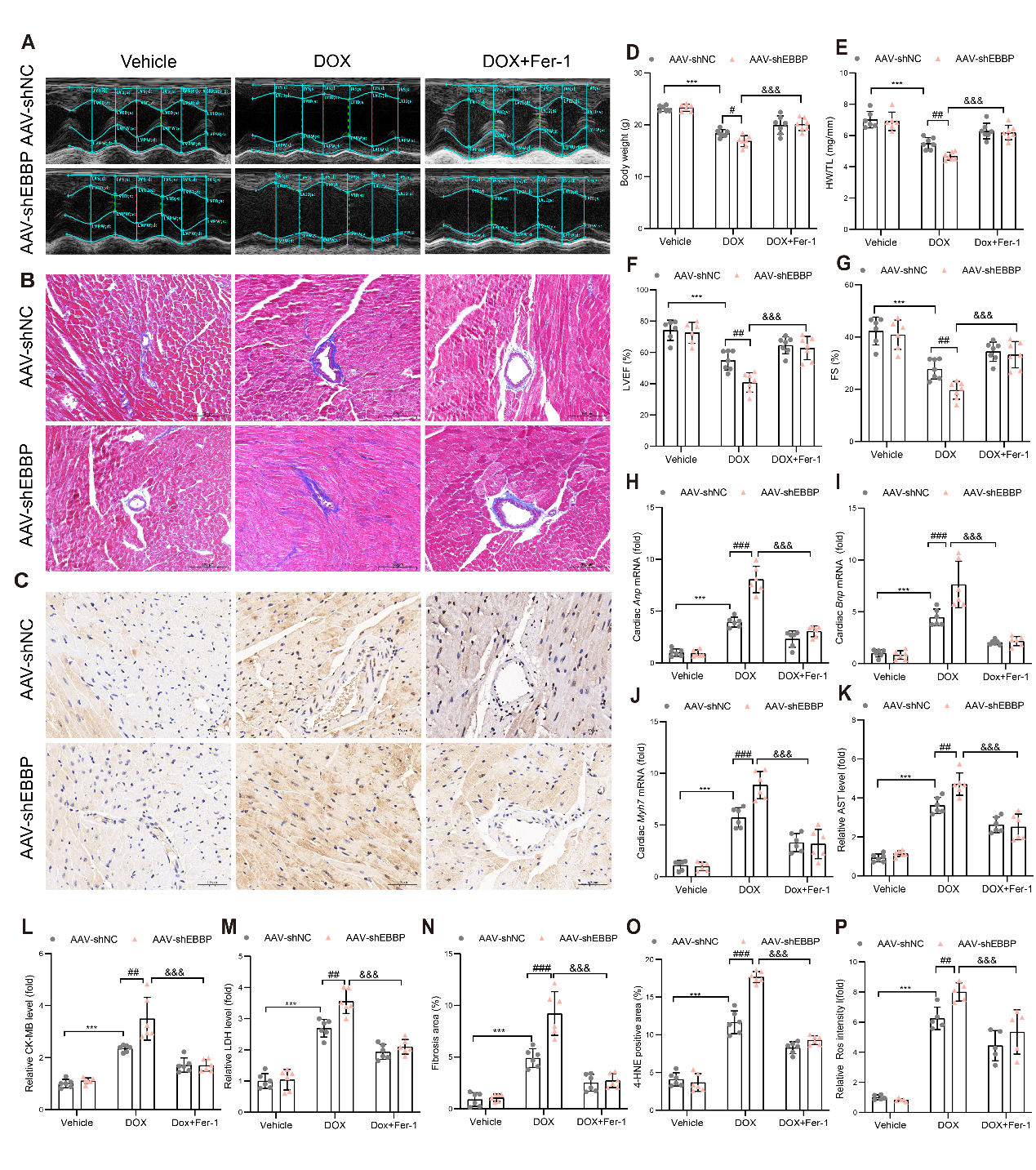


**FigureS13. Fer-1** **abrogates the detrimental effect of EBBP deficiency on anthracycline-induced cardiotoxicity.**

AAV-shNC or AAV-shEBBP was administered via tail vein injection, and the DoIC model was established 14 days thereafter. To inhibit ferroptosis, mice also received a daily injection of Fer-1 during the DOX treatment.

**A.** Representative images of transthoracic echocardiography. **B.** Representative images of Masson’s trichrome staining (scale bars, 100μm). **C.** Representative images of immunohistochemistry for 4-HNE in mouse myocardium (scale bars, 50μm). **D.** Changes in body weight of mice (n = 6). **E.** Changes in HW/TL (n=6). **F-G.** Statistical analysis of LVEF and FS (n=6). **H-J.** The mRNA levels of Anp, Bnp, and Myh7 in cardiac tissues (n=6). **K-M.** Statistical analysis of serum AST, CK-MB, and LDH levels (n=6). **N.** Statistical analysis of fibrosis area in mouse myocardium (n=6). **O.** Statistical analysis of immunohistochemistry for 4-HNE in mouse myocardium(n=6). **P.** Quantification of fluorescent immunohistochemistry staining for DHE in mouse myocardium (scale bars, 100μm, n=5). Values are presented as the mean±SD. ****p* < 0.001 vs. Vehicle + AAV-shNC; ^#^*p* < 0.05, ^##^*p* < 0.01 and ^###^*p* < 0.001 vs. DOX + AAV- shNC group; ^&&&^*p* < 0.001 vs. DOX + AAV-shEBBP group.


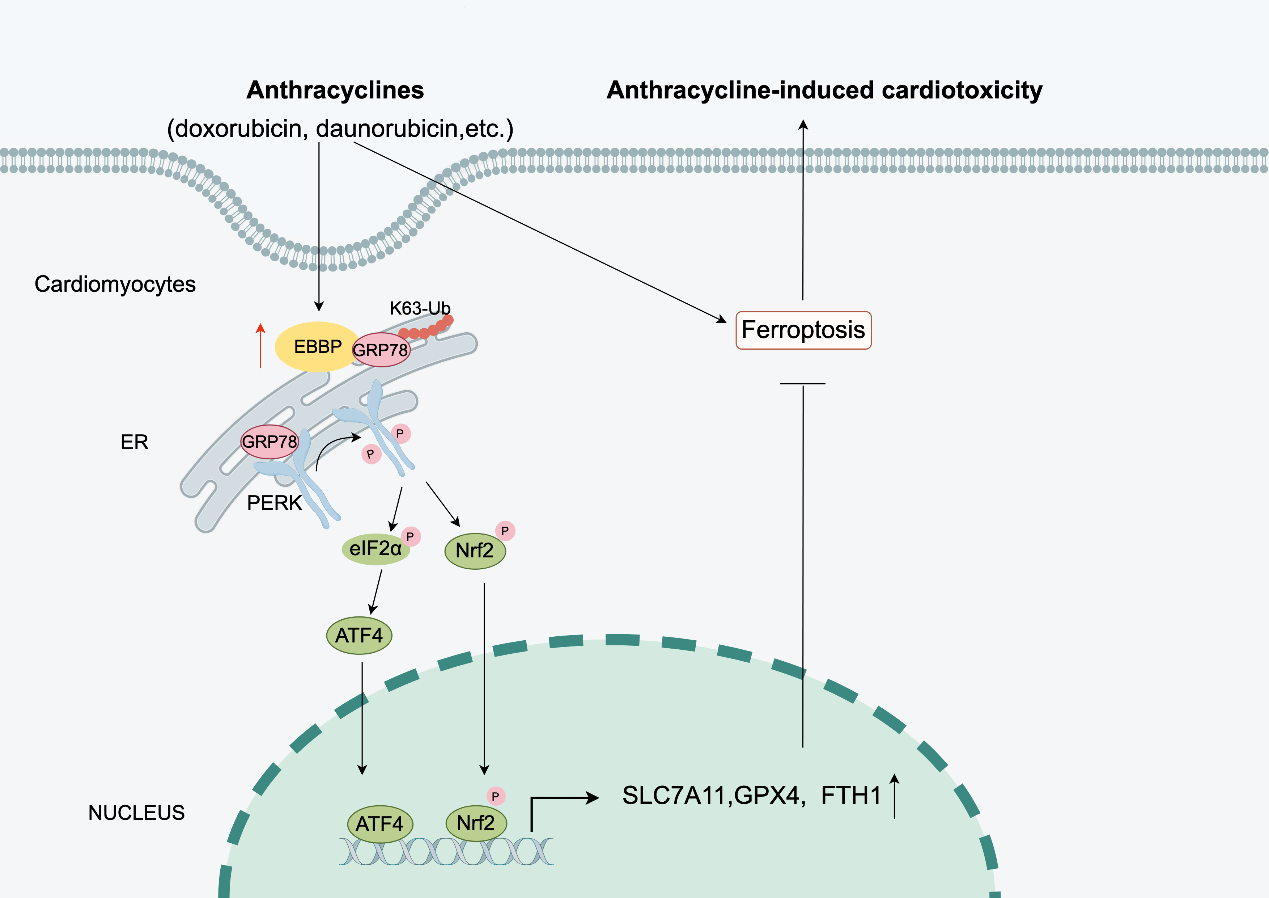


**Figure S14. Mechanism diagram of EBBP in DOX-induced cardiotoxicity.**

EBBP expression is significantly upregulated in DOX-treated cardiomyocytes. Mechanistically, EBBP interacts with GRP78 to mediate K63-linked ubiquitination, thereby attenuating the inhibitory GRP78-PERK interaction and triggering activation of the PERK-mediated integrated stress response (ISR). This signaling cascade culminates in the activation of downstream effectors ATF4 and Nrf2, which transcriptionally upregulate the SLC7A11-GSH-GPX4 antioxidant axis and concurrently alleviate iron overload.

**Supplemental table 1. Primary antibodies used in this study.**

| **Reagents** | **Source** | **Catalogue number** |
| --- | --- | --- |
| EBBP | Invitrogen | PA5-104489 |
| EBBP | Affinity | AF0352 |
| GPX4 | Proteintech | 67763-1-lg |
| Myc | Proteintech | 16286-1-AP |
| SLC7A11/xCT | Proteintech | 26864- 1-AP |
| FTH1 | Abclonal | A19544 |
| ATF4 | Proteintech | 60035-1-Ig |
| eIF2α | Abclonal | A0764 |
| p-eIF2α-S49 | Abclonal | AP0341 |
| PERK | Abclonal | A18196 |
| p-PERK | Abclonal | AP1501 |
| Nrf2 | Abclonal | A12446 |
| p-Nrf2 | Abclonal | AP1133 |
| GAPDH | Proteintech | 60004-1-Ig |
| GRP78 | Proteintech | 11587-1-AP |
| Flag | Sigma Aldrich | F1804 |
| HA | Proteintech | 51064-2-AP |
| Ub | Proteintech | 10201-2-AP |
| p-RIPK3 | Affinity | AF7443 |
| RIPK3 | Abclonal | A5431 |
| p-MLKL | Affinity | AF7420 |
| MLKL | Abclonal | A5579 |
| p-RIPK1 | Affinity | AF2398 |
| RIPK1 | Abclonal | A7414 |
| ACSL4 | Proteintech | 22401-1-AP |
| GCLM | Proteintech | 14241-1-AP |
| GCLC | Proteintech | 12601-1-AP |
| NQO1 | Proteintech | 67240-1-ig |
| HO-1 | Proteintech | 10701-1-AP |
| Parkin | Proteintech | 14060-1-AP |
| PINK1 | Proteintech | 23274-1-AP |
| ATG7 | Proteintech | 10088-2-AP |
| LC3B | CST | 2775 |
| P62 | Proteintech | 18420-1-AP |
| Caspase1 | Affinity | AF5418 |
| Cleaved-Caspase1 | Affinity | AF4005 |
| GSDMD | Affinity | AF4012 |
| Cleaved-Caspase3 | Affinity | AF7022 |
| NLRP3 | Abclonal | A5652 |
| BAX | Proteintech | 50599-2-Ig |
| Bcl2 | Proteintech | 26593-1-AP |

**Supplemental table 2. Primers for real-time PCR detection.**

| **Primers** | **Sequences 5'---3'** |
| --- | --- |
| Human *EBBP* Fw | GTCCTGTCTAACCTGCATGGT |
| Human *EBBP* Re | GGCAGTATCGCCAGTTGTG |
| Rat *EBBP* Fw | CACCAGCAGTGTATCTGCCA |
| Rat *EBBP* Re | ACCGAAGGTCAGCCTCTTTG |
| Mouse *EBBP* Fw | TCTTGGGGCCAGCAGAGTAA |
| Mouse *EBBP* Re | CTCACAGTAGTTCACCATGCAG |
| Mouse *Anp* Fw | GGATTTCAAGAACCTGCTAGACC |
| Mouse *Anp* Re | GATCTATCGGAGGGGTCCCA |
| Mouse *Bnp* Fw | AGTCCTTCGGTCTCAAGGCA |
| Mouse *Bnp* Re | CAACAACTTCAGTGCGTTACAGC |
| Mouse *Myh7* Fw | CTACCCTCAGGTAGGAGTGGG |
| Mouse *Myh7* Re | TTTCTCGGAGCCACCTTGGAA |
| Mouse *Ptgs2* Fw | TGAGCAACTATTCCAAACCAGC |
| Mouse *Ptgs2* Re | GCACGTAGTCTTCGATCACTATC |
| Rat *Ptgs2* Fw | TCCTTGCTGTTCCAACCCAT |
| Rat *Ptgs2* Re | TTCTTGTCAGAAACTCAGGCGT |
| Mouse *18s* Fw | GCACCACCACCCACGGAATCG |
| Mouse *18s* Re | TTGACGGAAGGGCACCACCAG |
| Rat *Nqo1* Fw | AGAAACGACATCACAGGGGAG |
| Rat *Nqo1* Re | GCGAATCCTGCTACAAGCAC |
| Rat *Gclc* Fw | GAAGAGACCCAGCGCCAC |
| Rat *Gclc* Re | ACATGTACTCCACCTCGTCAC |
| Rat *Gclm* Fw | CACAATGACCCAAAAGAACTGCT |
| Rat *Gclm* Re | CGCTTTTTGTAGGCGAGAGC |
| Rat *Hmox1* Fw | CCACGCATATACCCGCTACC |
| Rat *Hmox1* Re | AGCTCCTCAAACAGCTCAATGT |
| Rat *Cth* Fw | GGCCAGTCCTCGGGTTTTG |
| Rat *Cth* Re | AGTCCAAACTCGGATGCCAC |
| Rat *Psph* Fw | CTCATCTCCGGGGGCTTTAG |
| Rat *Psph* Re | CAATGAAGGCATCAGCGGGAG |
| Rat *Psat1* Fw | TGGCATCAGTGTGCTTGAAATG |
| Rat *Psat1* Re | ACTTCTTGGCTTCTTCCGCA |
| Rat *Slc1A4* Fw | GTCGTGGATCATGTGGTACG |
| Rat *Slc1A4* Re | GCTGGAACAGGTCGCAAAAG |
| Rat *Slc1A5* Fw | GGGCCCTGCTCTTTTTCCT |
| Rat *Slc1A5* Re | GACAGGCACCACATGGAATG |
| Mouse *Mphosph1* Fw | AGACTCTGGAACGGTGTCTGA |
| Mouse *Mphosph1* Re | AGTTGTTGCCAAATCTGCTCAT |
| Mouse *Slc43a2* Fw | TGCACCGCTGTGTTGGAAA |
| Mouse *Slc43a2* Re | CCGTGCTGTTAGTGACATTCTC |
| Mouse *Il4ra* Fw | TCTGCATCCCGTTGTTTTGC |
| Mouse *Il4ra* Re | GCACCTGTGCATCCTGAATG |
| Mouse *Rai14* Fw | CGATACAAACGAGTGGAACAAGA |
| Mouse *Rai14* Re | TCGCTGTCATGCTTCGTGG |
| Mouse *Msn* Fw | TCTTATGCCGTCCAGTCTAAGT |
| Mouse *Msn* Re | GGTCCTTGTTGAGTTTGTGCT |
| Mouse *Csf3R* Fw | CTGATCTTCTTGCTACTCCCCA |
| Mouse *Csf3R* Re | GGTGTAGTTCAAGTGAGGCAG |
| Mouse *Fpr1* Fw | CATTTGGTTGGTTCATGTGCAA |
| Mouse *Fpr1* Re | AATACAGCGGTCCAGTGCAAT |
| Mouse *Ebbp* Fw | TGGCACCTATGTTGGCCTG |
| Mouse *Ebbp* Re | TGCAGTCAAACTTGTGAATCAGA |
| Mouse *Adamts7* Fw | GCAGGCTTCGTCTGCTTTCTA |
| Mouse *Adamts7* Re | GCCATCAGATAAGGGTTGGTGG |
| Mouse *Mrgprh* Fw | GTATCCTCTGGAATCCACACAAC |
| Mouse *Mrgprh* Re | AGGGAACAAATGACCAGGGAG |
| Mouse *Pm20D1* Fw | CTTCTCTTTTTCGCTACGGTCT |
| Mouse *Pm20D1* Re | CACCTTTCAGCGCCTCTTTTAT |
